# Fruits of *Hippophaë rhamnoides* in human leukocytes and Caco-2 cell monolayer models—A question about their preventive role in lipopolysaccharide leakage and cytokine secretion in endotoxemia

**DOI:** 10.3389/fphar.2022.981874

**Published:** 2022-09-30

**Authors:** Anna K. Laskowska, Aleksandra Wilczak, Weronika Skowrońska, Piotr Michel, Matthias F. Melzig, Monika E. Czerwińska

**Affiliations:** ^1^ Department of Pharmaceutical Microbiology, Faculty of Pharmacy, Medical University of Warsaw, Warsaw, Poland; ^2^ Centre for Preclinical Research, Medical University of Warsaw, Warsaw, Poland; ^3^ Student Scientific Association, Department of Pharmacognosy and Molecular Basis of Phytotherapy, Faculty of Pharmacy, Medical University of Warsaw, Warsaw, Poland; ^4^ Department of Pharmacognosy and Molecular Basis of Phytotherapy, Faculty of Pharmacy, Medical University of Warsaw, Warsaw, Poland; ^5^ Department of Pharmacognosy, Faculty of Pharmacy, Medical University of Lodz, Lodz, Poland; ^6^ Institute of Pharmacy, Freie Universitaet Berlin, Berlin, Germany; ^7^ Department of Biochemistry and Pharmacogenomics, Faculty of Pharmacy, Medical University of Warsaw, Warsaw, Poland

**Keywords:** epithelium, gut barrier leakage, low-grade inflammation, metabolic endotoxemia, sea buckthorn

## Abstract

Preparations from *Hippophaë rhamnoides* L. (sea buckthorn) have been traditionally used in the treatment of skin and digestive disorders, such as gastritis, gastric and duodenal ulcers, uterine erosions, as well as oral, rectal, and vaginal mucositis, in particular in the Himalayan and Eurasian regions. An influence of an aqueous extract from the fruits of *H. rhamnoides* (HR) on leakage of lipopolysaccharide (LPS) from *Escherichia coli* through gut epithelium developed from the human colorectal adenocarcinoma (Caco-2) monolayer *in vitro* and glucose transporter 2 (GLUT2) translocation were the principal objectives of the study. Additionally, the effect of HR on the production of pro- and anti-inflammatory cytokines (interleukins: IL-8, IL-1*β*, IL-10, IL-6; tumor necrosis factor: TNF-*α*) by the Caco-2 cell line, human neutrophils (PMN), and peripheral blood mononuclear cells (PBMC) was evaluated. The concentration of LPS on the apical and basolateral sides of the Caco-2 monolayer was evaluated with a Limulus Amebocyte Lysate (LAL) assay. GLUT2 translocation was evaluated using an immunostaining assay, whereas secretion of cytokines by cell cultures was established with an enzyme-linked immunosorbent (ELISA) assay. HR (500 μg/ml) significantly inhibited LPS leakage through epithelial monolayer *in vitro* in comparison with non-treated control. The treatment of Caco-2 cells with HR (50–100 μg/ml) showed GLUT2 expression similar to the non-treated control. HR decreased the secretion of most pro-inflammatory cytokines in all tested models. HR might prevent low-grade chronic inflammation caused by metabolic endotoxemia through the prevention of the absorption of LPS and decrease of chemotactic factors released by immune and epithelial cells, which support its use in metabolic disorders in traditional medicine.

## 1 Introduction

Fruits and vegetables are easily accessed plant materials, which provide health beneficial effects. For this reason, they are often recommended in dietary guidelines to reduce the risk of chronic disorders (Ma X. et al., 2022). A folk functional food concept has a long history of usage in Asian populations. Sea buckthorn (*Hippophaë rhamnoides* L., Elaeagnaceae) is native to the Himalayan region and distributed across Eurasia, in particular in the cold-temperate regions like seashores and mountains of Asia (Central Asia, India, China, Tibet, Mongolia, the Caucasus, and Turkey) as well as Northwestern Europe. Nowadays, it is cultivated in orchards, particularly in Europe, Canada, and the United States (Pundir et al., 2021; Ma Z. et al., 2022). Both leaves and fruits of sea buckthorn are a rich source of phytochemicals. In particular, fruits provide dietary products such as juice from the fleshy tissue of berries and oil produced from their seeds. The application of *H. rhamnoides* fruits (*Shaji*) in traditional Chinese medicine involved probiotic jams, and beverages, or the fruits were directly eaten due to their anti-tumor properties as well as supporting cerebral vessels, cardiovascular and digestive systems (Gong et al., 2020). Sea buckthorn berries are used traditionally for treating cough, asthma, hypertension, rheumatism, and jaundice. Infusions are often prepared for alleviation of skin diseases and digestive disorders. The oil extracted from berries is used for the treatment of gastric and duodenal ulcers, gastritis, and uterine erosions. Additionally, the oil is known for its beneficial effects in the oral, rectal, and vaginal mucositis. The plant materials of sea buckthorn are also reported in the traditional formulations used for improving blood circulation and digestion, as well as in constipation, burns, and wound healing (Li, 1999; Singh et al., 2014). The dermatological and pharmacological significance of *H. rhamnoides* has been recently revised (Pundir et al., 2021).

In addition to carotenoids and vitamins, the *H. rhamnoides* fruits are a rich source of polyphenolic compounds, particularly flavonoids such as derivatives of isorhamnetin, quercetin, and kaempferol (Fang et al., 2013; Ciesarová et al., 2020; Jia et al., 2020). It is worth noting that both aglycones and glycosides of flavonols are effectively absorbed in animals (Hollman et al., 1999; Kammalla et al., 2015; Xie et al., 2015; Duan et al., 2016). A recent meta-analysis has provided evidence that dietary intake of sea buckthorn berries or their extracts significantly improved total cholesterol, triacylglycerol (TAG), low-density lipoprotein (LDL), and high-density lipoprotein (HDL) cholesterol in subjects involved in clinical trials (Guo et al., 2017). The aqueous extract from seeds of sea buckthorn exhibited hypoglycemic activity and its activity enhanced insulin sensitivity (Zhang et al., 2010). Flavonoids were indicated as bioactive constituents responsible for the reduction of blood glucose concentration, as well as levels of fatty acids and blood lipids. It was established that flavonoids protected *β* cells in pancreatic islets and prevented lipid peroxidation (Gong et al., 2020). In our previous study, the digested fractions obtained from the aqueous extract of *H. rhamnoides* fruits (HR), rich in isorhamnetin derivatives, inhibited the activity of pancreatic lipase and *α*-amylase *in vitro* (Siegień et al., 2021).

Injuries of intestinal barrier are considered a risk of metabolic endotoxemia, which is caused by the increase of lipopolysaccharide (LPS) in plasma. Next, increasing concentration of LPS is associated with systemic low-grade inflammation. This pathological state leads to the development of a wide range of chronic disorders such as type 2 diabetes mellitus, non-alcoholic fatty liver disease, and cardiometabolic diseases like atherosclerosis (Mohammad and Thiemermann, 2020). An increase in intestinal LPS-bearing bacterial species initiates the cascade of the inflammatory response through subsequent activation of Toll-like receptors (TLRs) on immune cells. Lipopolysaccharide is considered the principal pathogen-associated molecular pattern (PAMP) in Gram-negative bacteria (Sampath, 2018).

A model of human colorectal adenocarcinoma (Caco-2) cells is one of the useful tool for evaluation of intestinal permeability *in vitro*. Caco-2 cells spontaneously differentiate to form confluent monolayer which both structurally and functionally resembles the small intestinal epithelium (Hilgers et al., 1990). The mucosal protectors, e.g. sucralfate, are used both in the treatment of peptic ulcer disease and infectious diarrhea (Lopetuso et al., 2015). Apart from sucralfate, gelatin tannate, a complex of tannic acid and gelatine, is a useful protector of a gut barrier. It develops a protective film and forms bonds with mucin (Ruszczyński et al., 2014). Thus, it protects the gut barrier against the influence and penetration of commensal bacteria or their metabolites (Lopetuso et al., 2015). A key nutritional aspect of the mucus layer is its high polysaccharide content. It is known that dietary fiber protects the intestinal barrier physically by enhancing the colonic mucus barrier. Therefore, the arising question is if other compounds might play a similar role (Desai et al., 2016).

The aim of this study was an assessment of the potential inhibition of leakage of LPS from *Escherichia coli* through gut epithelium developed from the Caco-2 monolayer *in vitro* by the HR extract. Additionally, the effect of the extract on GLUT2 translocation in Caco-2, as well as the production of pro- and anti-inflammatory cytokines (interleukins: IL-8, IL-1*β*, IL-10, IL-6; tumor necrosis factor: TNF-*α*) by the Caco-2 cells, human neutrophils (PMN), and peripheral blood mononuclear cells (PBMC) was evaluated.

## 2 Materials and methods

### 2.1 Experimental reagents

Fruits of *Hippophaë rhamnoides* were supplied by Zakład Konfekcjonowania Ziół FLOS (Mokrsko, Poland; batch no. 1058). Pierce LAL (Limulus Amebocyte Lysate) Chromogenic Endotoxin Quantitation Kit (88282) was obtained from Thermo Fisher Scientific (Rockford, IL, United States). Glut2 polyclonal antibody (600-401-GN3, Rockland, PA, United States), FITC-conjugated goat anti-Rabbit IgG (H + L) Cross-Adsorbed Secondary Antibody (F-2765, Invitrogen, United Kingdom), and 4′,6-diamidino-2-phenylindole dihydrochloride (DAPI; D1306) were purchased from Thermo Fisher Scientific (Rockford, IL, United States). Phosphate buffered saline (PBS, L0615) and Dulbecco’s Modified Eagle Medium (DMEM, L0103), fetal bovine serum (FBS, S1860) as well as penicillin-streptomycin (L0022) were purchased from Biowest (Nauillé, France). Pancoll Human (P04-601000) was from PAN-Biotech (Aidenbach, Germany). TrypLE^™^ Express Enzyme (12604021) was purchased from Gibco Thermo Fisher (Waltham, MA, United States). Citric acid (CHEM-115382101), sodium citrate tribasic dihydrate (C7254), glucose (114595600) for citrate dextrose solution (ACD) were purchased from Chempur (Piekary Śląskie, Poland). Dextran from *Leuconostoc mesenteroides* (31398), propidium iodide (PI, P4170-10MG), dexamethasone (Dex, D1756), sodium butyrate (303410), Triton X-100 (T8787), and bovine serum albumin (BSA; A9418) were purchased from Sigma-Aldrich (Saint Louis, MO, United States). RPMI 1640 medium (R7509), amphotericin B (A2942), and 3-(4,5-dimethylthiazol-2-yl)-2,5-diphenyltetrazolium bromide (MTT, 0793) were purchased from Sigma-Aldrich (Saint Louis, MO, United States). Recombinant human IL-1*β* protein (IL038), LPS (2552117) from *Escherichia coli* (0111:B4), and formic acid (100264) were obtained from Merck (Darmstadt, Germany). Recombinant TNF-*α* (300-01A), as well as recombinant interferon (IFN)-*γ* (300-02), were purchased from PeproTech (United States). Sets of immunosorbent assays for human IL-8 (555244), TNF-*α* (555212), IL-1*β* (557953), IL-6 (555220), and IL-10 (555157) were purchased from BD Biosciences (Erembodegem, Belgium). Pierce BCA Protein Assay Kit (23225) was obtained from Thermo Fisher Scientific (Rockford, IL, United States). The Caco-2 cell line originated from the German Collection of Microorganisms and Cell Cultures (DSMZ), Leibnitz Institute (Braunschweig, Germany). Buffy coats, used for PMN and PBMC isolation, were purchased from the Warsaw Blood Donation Centre (Warsaw, Poland).

### 2.2 Extract preparation

Preparation of the aqueous extracts of the *H. rhamnoides* fruits: 5 g of powdered plant material was extracted three times with boiling water in a ratio of 1:40 (*m*/*v*) for 15 min. The collected aqueous extracts were concentrated under reduced pressure (Rotavapor R-3, Buchi, Switzerland) and lyophilized (Telstar Cryodos 50, Terrassa, Spain). The preparation of extracts for isolation procedures was provided in the Supplementary Material.

### 2.3 Caco-2 cell culture

The Caco-2 cells were cultured in DMEM supplemented with 20% FBS (*v*/*v*), L-alanyl-L-glutamine (2 mM), penicillin (100 IU/ml), streptomycin (100 μg/ml), and amphotericin B (2.5 ng/ml). The details of cell culturing were previously described (Czerwińska et al., 2021). Passages between 20 and 45 were used in all *in vitro* experiments. For experiments, the cells were seeded in 24-well plates at a density of 2×10^5^ cells/well in DMEM with a 20% FBS (*v*/*v*). After 24 h the procedure of differentiation (72 h) was started by replacing the complete medium with DMEM supplemented with 5% FBS (*v*/*v*) and sodium butyrate (5 mM) (Czerwińska et al., 2021).

#### 2.3.1 LPS concentration in the apical and basolateral compartment of Caco-2 monolayer

For the evaluation of LPS leakage from the apical to basolateral side, the Caco-2 cells were plated at a density of 2 × 10^4^ cells/cm^2^ on polycarbonate membranes with a pore size of 0.4 µm and pore density of 1 × 10^8^/cm^2^ in 12-well translucent ThinCert^™^ (Biokom, Janki, Poland). The cells were maintained for 21–23 days at 37°C in a humidified atmosphere of 95% air and 5% CO_2_. The growth medium was replaced every 2–3 days until the cells were fully differentiated and formed a monolayer. The integrity of the monolayer was monitored by transepithelial electrical resistance (TEER) measurements using the Millicell Electrical Resistance System (Millipore, Merck KGaA, Darmstadt, Germany). Only monolayers with TEER values above 500 Ω × cm^2^ were used in experiments. The cells were treated with the HR extract at a concentration of 500, 1000, and 2000 μg/ml. After 30 min LPS (100 ng/ml) was added to the apical side of the Caco-2 monolayer for further 24 h incubation. LAL Chromogenic Endotoxin Quantitation Kit was used to determine LPS concentration in the apical and basolateral compartments according to the manufacturer’s protocol. Briefly, bacterial endotoxin catalyzes the activation of a proenzyme in the modified LAL test, which next splits *p*-nitroaniline (*p*NA) from the colorless substrate. The released *p*NA was photometrically measured at 405 nm in a microplate reader (Synergy 4, Biotek, Winooski, VT, United States). The developed color intensity is proportional to the amount of endotoxin present in the sample and can be calculated using a standard curve linear in the 0.1–1.0 EU/ml range. The results were expressed per mg of the protein.

#### 2.3.2 IL-8 secretion by Caco-2 cells

The differentiated Caco-2 cells were cultured overnight in the absence or presence of the HR extract at concentrations of 5, 50, and 100 μg/ml for 24 h. Then, the cells were stimulated for the next 24 h with the cocktail composed of IL-1*β* (25 ng/ml), TNF-*α* (10 ng/ml), IFN-*γ* (10 ng/ml), and LPS (100 ng/ml) for IL-8 secretion. The released into supernatants IL-8 was measured by enzyme-linked immunosorbent assay (ELISA) following the indications of the manufacturer. The concentration of protein content was measured with BCA Protein Assay Kit. Dexamethasone (Dex) in the concentrations of 5, 25, and 100 µM was used as a positive control.

The viability of differentiated Caco-2 cells treated with the HR extract was measured with the MTT assay as described previously (Czerwińska et al., 2021). The positive control was a 10% solution of Triton X-100 (*v*/*v*). The reagent solution of MTT (0.5 mg/ml) was replaced with DMSO, and absorbance was measured at 560 nm (test) and 620 nm (reference) in the microplate reader (Synergy 4, Biotek, Winooski, VT, United States).

#### 2.3.3 GLUT2 translocation

The differentiated Caco-2 cells were treated with the HR extract at concentrations of 50, 100, 500, 1000, and 2000 μg/ml for 24 h. The glucose-reduced medium was prepared by 4-fold diluting of the culture medium enriched with glucose at a concentration of 4.5 g/L. Phloretin (20 µM) was used as a positive control. Glucose solutions at a concentration of 50 and 75 mM were used as negative controls (Cohen et al., 2014). The differentiated Caco-2 cells, which were treated with extracts or controls, were fixed with a 4% paraformaldehyde (PFA) solution for 20 min at room temperature. Next, the cells were washed with PBS for 5 min. Blocking of nonspecific bonds was carried out for 60 min in a 3% solution of BSA in PBS. Subsequently, cells were incubated with the primary polyclonal antibody GLUT2 (5 μg/ml) for 60 min at room temperature. After incubation, the cells were washed 3 times with PBS and incubated with FITC-conjugated secondary antibody (1: 400) for 45 min at room temperature in the darkness. To label the cell nuclei, cells were incubated with DAPI (10 μg/ml) for 5 min at room temperature. GLUT2 receptors were visualized with a fluorescence microscope (Nikon Eclipse TR100, Nikon^®^ Nikon Instruments Inc., Melville, NY, United States) supported by NIS-Elements 2.0 software.

### 2.4 Isolation of PMN

Neutrophils were isolated by dextran sedimentation and centrifugation in a density gradient with buffer for leukocytes isolation (Pancoll Human, 1.077 g/ml) according to the Böyum’s method (Böyum, 1968). RPMI 1640 medium with stable L-glutamine, 25 mM HEPES, 10% FBS, penicillin (100 U/ml), and streptomycin (100 μg/ml) solution was used to suspend the cells.

The viability of PMN was routinely evaluated with PI (0.5 μg/ml) staining using flow cytometry with FACS Calibur (BD Biosciences, San Jose, CA, United States) as described previously (Czerwińska et al., 2021). The cells were treated with extracts at concentrations of 5, 50, and 100 μg/ml for 24 h. Triton X-100 (0.1%, *v*/*v*) was used as a positive control.

The cytokine secretion by LPS-stimulated (100 ng/ml) PMN was determined with ELISA. The ELISA assays for released TNF-*α*, IL-8, and IL-1*β* into cell culture supernatants were performed by following the indications of the manufacturer. The effect on TNF-*α*, IL-8, and IL-1*β* secretion was calculated as the percentage of released cytokine in comparison with stimulated control without tested extract (+LPS). Dexamethasone was used as the positive control.

### 2.5 Isolation of PBMC

The buffy coats fractions (40 ml) were diluted with PBS. The blood was covered with a layer of equal quantity of Pancoll Human (density 1.077 g/ml) and centrifuged (2200 RPM, 20 min, 25°C). Mononuclear cells were carefully harvested by aspiration. Finally, RPMI 1640 medium with stable L-glutamine, 25 mM HEPES, 10% FBS, penicillin (100 U/ml), and streptomycin (100 μg/ml) solution was used to suspend the cells.

The viability of PBMC was routinely evaluated with PI (0.5 μg/ml) staining using flow cytometry with FACS Calibur (BD Biosciences, San Jose, CA, United States) as described previously (Olszewska et al., 2020). The cells were treated with extracts at concentrations of 5, 50, and 100 μg/ml for 24 h. Triton X-100 (0.1%, *v*/*v*) was used as a positive control.

The cytokine secretion by LPS-stimulated (100 ng/ml) PBMC was determined with ELISA. The ELISA assays for released TNF-*α*, IL-6, and IL-10 into cell supernatants were performed by following the indications of the manufacturer. The effect on TNF-*α*, IL-6, and IL-10 production was calculated as the percentage of released cytokine in comparison with stimulated control without tested extract (+LPS). Dexamethasone was used as the positive control.

### 2.6 Chromatographic analysis

Chromatographic analysis was performed on a UHPLC-3000 RS system (Dionex, Germany) with DAD and an AmaZon SL ion trap mass spectrometer with an ESI interface (Bruker Daltonik GmbH, Germany). Separations were performed on a Kinetex XB-C_18_ (150 × 2.1 mm, 1.7 μm) column (Phenomenex Inc., Torrance, CA, United States). The column oven temperature was maintained at 25°C. For preliminary phytochemical analyses of the extracts and fractions, mobile phase A was 0.1% HCOOH in water, and mobile phase B was 0.1% HCOOH in acetonitrile. The gradient program was as follows: 0–10% B, 5–15 min; 10–20% B, 15–25 min; 20–30% B, 25–35 min; 30–50% B, 35–45 min; 50–100% B, 45–50 min; 100% B, 50–60 min; 0%B, 60–70 min (equilibration). The flow rate was 0.2 ml/min. UV spectra were recorded 200—800 nm range, and chromatograms were acquired at 240, 280, 325 nm, or 520 nm. The LC eluate was introduced directly into the ESI interface without splitting. The details were previously described (Świerczewska et al., 2019).

The isolation procedures of flavonoids as well as their NMR spectra were provided in the Supplementary Figures S1–S13.

### 2.7 Total polyphenol content

The sum of phenols was determined using a modified spectrophotometric method with Folin-Ciocalteu’s reagent (Singleton et al., 1999), according to the assay previously described (Czerwińska et al., 2016). The absorbance was measured at 765 nm in the microplate reader (SYNERGY 4, BioTek, Winooski, VT, United States). The results were expressed as gallic acid equivalent (µg GAE/mg d.w. of the extract).

### 2.8 Statistical analysis

The results were presented as a means ± standard error of the mean (S.E.M). All samples were studied in at least three independent experiments in triplicate. Statistical significance of the differences between means was evaluated by testing homogeneity of variance and normality of distribution followed by one-way ANOVA. The non-parametric methods such as Kruskal-Wallis or U-Mann-Whitney’s tests were used if necessary. *p* values below 0.05 were considered statistically significant. The results were analyzed using GraphPad Prism (GraphPad Software, San Diego, CA, United States) and Statistica 13.3 (TIBCO Software Inc., Palo Alto, CA, United States) softwares.

## 3 Results

### 3.1 Inhibition of LPS efflux

**FIGURE 1:**
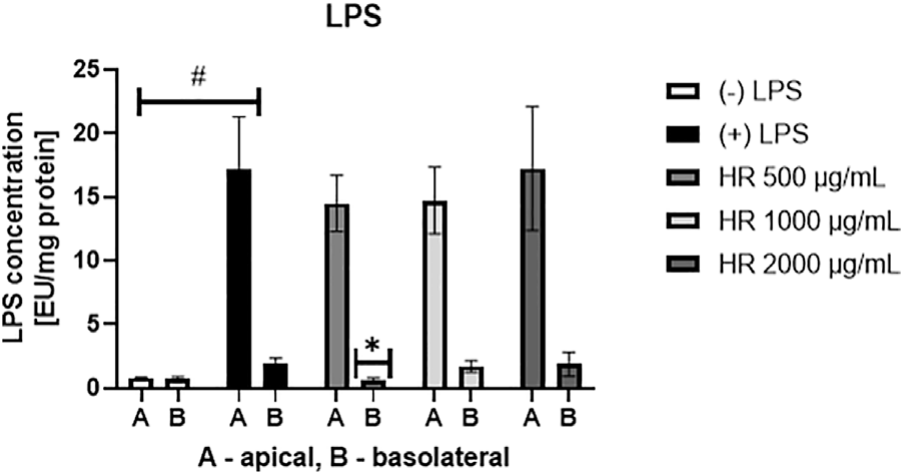
Concentration of LPS [EU/mg protein] in the apical and basolateral side of Caco-2 monolayer after the HR application. HR—aqueous extract from the fruit of *Hipopphaë rhamnoides*. ^#^ *p* < 0.001 vs. (-)LPS (A, apical side); *p <0.05 vs. (+)LPS (B, basolateral side).

In the LAL assay, we assessed the concentration of LPS both in the apical and basolateral compartment of the Caco-2 monolayer (Figure 1). On the apical side, the concentration of LPS showed a tendency to decrease when the cells were treated with the HR extract at a concentration of 500 (*p* = 0.44) and 1000 μg/ml (*p* = 0.60), whereas in the LPS-treated samples of HR extract in the highest concentration LPS concentration was similar to only LPS-treated control. The HR extract at the concentration of 500 μg/ml significantly inhibited the efflux of LPS from the apical to the basolateral side (*p* <0.05) when compared with LPS-treated control. The higher concentrations of extracts did not influence significantly LPS efflux.

### 3.2 Effect of HR extract on IL-8 secretion by Caco-2 cells

**FIGURE 2:**
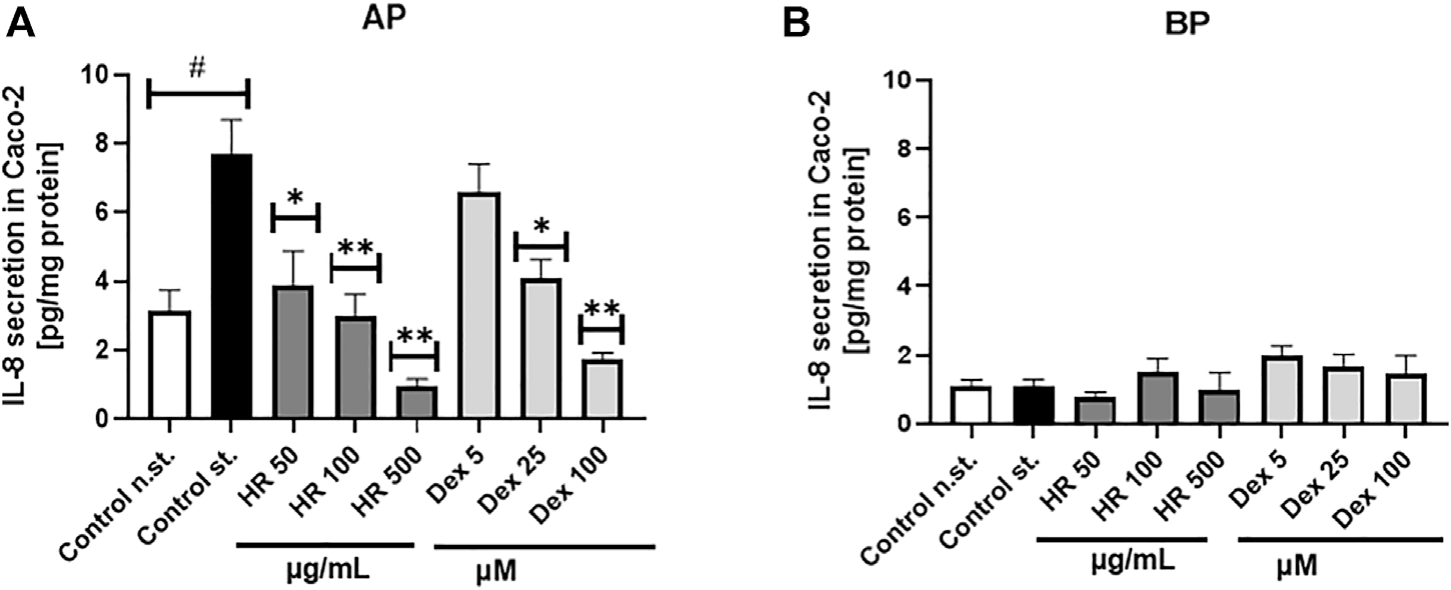
Effect of HR on IL-8 secretion by Caco-2 (pg/mg protein ± SEM) in the apical **(A)** and basolateral **(B)** side of Caco-2 monolayer after the HR application. HR—aqueous extract from the fruit of *Hipopphaë rhamnoides*; Dex—dexamethasone; st.—mixture of IL-1*β*/TNF-*α*/IFN-*γ*/LPS. ^#^ *p* < 0.001 vs. (−) st.; ^*^ *p* < 0.05, ^**^ *p* < 0.001 vs. (+) st.

The over-expression of IL-8 is observed in colonic tissues of patients suffering from ulcerative colitis. Interelukin-8 is considered a relevant chemoattractant and activator for immune cells. Chemoattracting of immune cells based on IL-8 gradient and their migration into the epithelium in addition to their stimulation is believed to deepen the injuries of epithelium (Wéra et al., 2016), which may determine an open gate for LPS leakage. The HR extract in the concentrations range between 50 and 500 μg/ml significantly inhibited IL-8 release to the apical side of the Caco-2 monolayer (Figure 2A). The effect of HR extract at a concentration of 500 μg/ml was stronger than Dex in the concentration range from 5 to 100 µM. On the other hand, we did not note any significant changes in the release of IL-8 to the basolateral side of the Caco-2 monolayer (Figure 2B). All tested samples showed concentrations of IL-8 similar to its concentration detected in the stimulatory cocktail-treated control (IL-1*β*/TNF-*α*/IFN-*γ*/LPS) on the basolateral side.

### 3.3 Effect of HR extract on cytokines secretion by PMN

**FIGURE 3:**
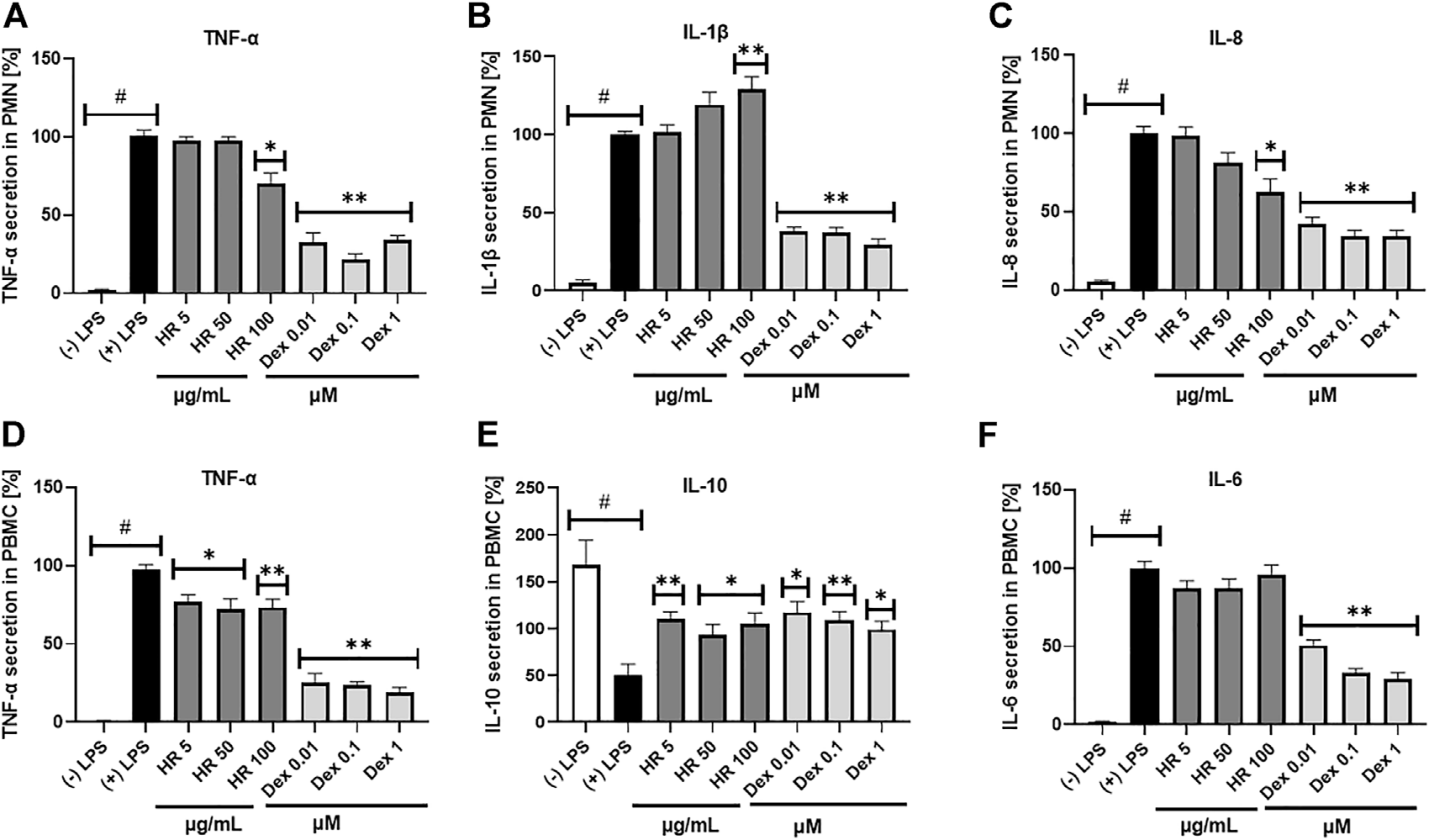
Effect of HR on secretion of cytokines (mean ± SEM [%]) by PMN **(A–C)** and PBMC **(D–F)**. HR—aqueous extract from the fruit of *Hipopphaë rhamnoides*; Dex—dexamethasone. ^#^ *p* <0.001 vs. (−) LPS; *p <0.05, **p <0.001 vs. (+) LPS.

Neutrophils can contribute to significant tissue damages during acute and chronic diseases when they are not properly eliminated and overstimulated. Metabolic alteration, such as diminished glucose-6-phosphate dehydrogenase (G6PD) and glutaminase activities, high free fatty acids, TAG, LPS, hyperglucose or hyperinsulin, are responsible for neutrophil dysfunctions in disease conditions (Neethi Raj et al., 2018; Kumar and Dikshit, 2019). The HR extract at a concentration of 100 μg/ml significantly inhibited the secretion of TNF-*α* and IL-8 by PMN (*p* <0.05), while it significantly increased the secretion of IL-1*β* (Figures 3A–C). The percentage of released TNF-*α* was 69.9 ± 7.0%, whereas IL-8 was 62.4 ± 8.7% in comparison with LPS-treated control (100.1 ± 4.3%). The effect of extract even in the highest concentration was weaker than the positive control of Dex in the case of TNF-*α* and IL-8 secretion. Due to the limited amount of isolated compounds 7, 10, and 11 we screened their effect on cytokine secretion only in PMN. In general, the compounds themselves did not significantly influence the secretion of cytokines (Supplementary Figure S14).

### 3.4 Effect of HR extract on cytokines secretion by PBMC

**FIGURE 4:**
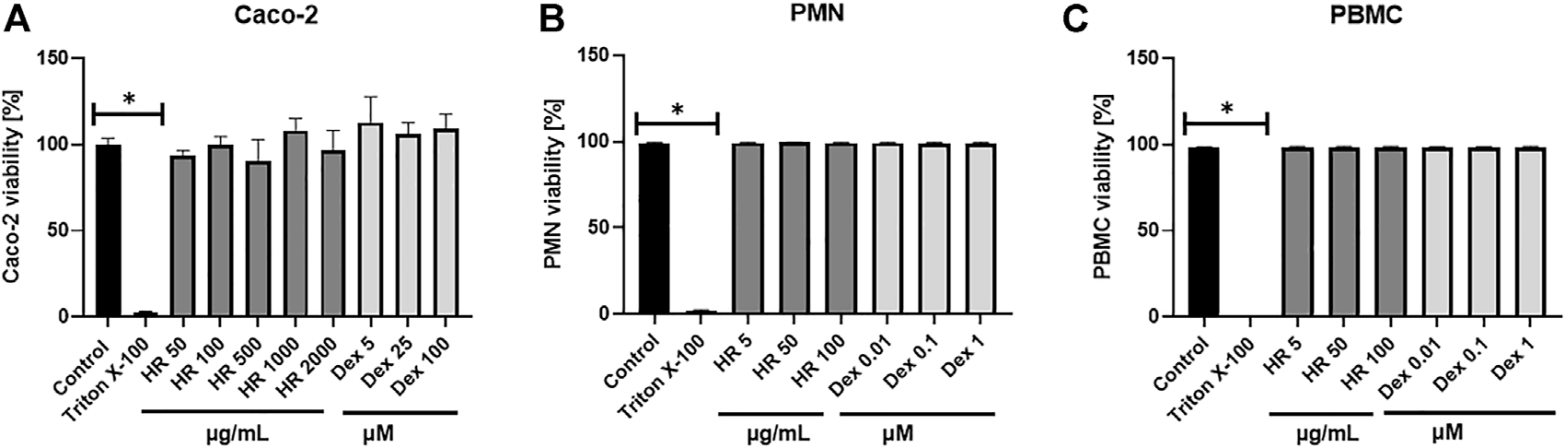
Effect of HR on cells viability **(A)** Caco-2 cells; **(B)** PMN; **(C)** PBMC. Data are expressed mean ± SEM [%]. Control—non-treated control cells; HR—aqueous extract from the fruit of *Hipopphaë rhamnoides*; Dex—dexamethasone. ^*^ *p* <0.001 vs. control.

Altering functions of PMN lead to the subsequent priming of PBMC. Their adherence, phagocytosis, chemotaxis and intracellular bactericidal functions are impaired or enhanced in diabetes mellitus (Neethi Raj et al., 2018). The extract significantly inhibited the secretion of TNF-*α* in the entire concentration range (Figure 3D). The percentage of released TNF-*α* ranged from 76.6 ± 4.8% for cells treated with 5 μg/ml HR (*p* <0.05) to 73.4 ± 5.2% for cells treated with 100 μg/ml HR (*p* <0.001) in comparison with LPS-treated control (97.2 ± 3.3%). The effect was not as relevant as in the case of Dex (*p* <0.001). Both HR extract and Dex in all tested concentrations increased the secretion of an anti-inflammatory cytokine such as IL-10 (Figure 3E). No effect of HR extract was observed on the release of IL-6 contrary to Dex (Figure 3F). The HR extract did not affect cells viability (Figure 4).

### 3.5 Inhibition of GLUT2 translocation in Caco-2 cells

**FIGURE 5:**
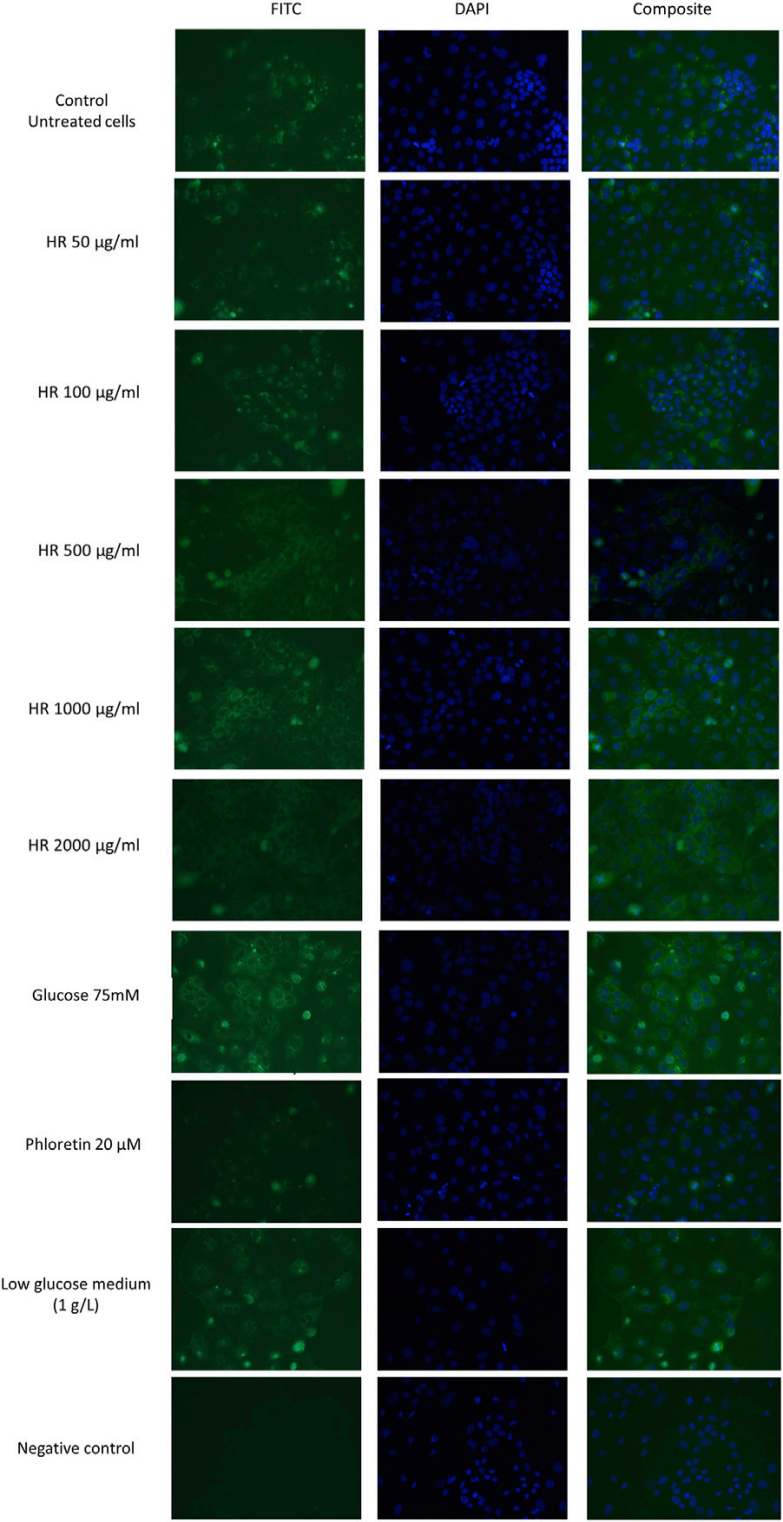
Detection of GLUT2 translocation in Caco-2 cells in a fluorescence microscope. HR—aqueous extract from the fruit of *Hipopphaë rhamnoides*.

GLUT2 mediates glucose homeostasis in the response of pancreatic *β* cells to rising glucose levels as well as in postprandial glucose uptake in the intestine and liver (Cohen et al., 2014). The results of our study were presented in Figure 5. In our study, phloretin, which is a dihydrochalcone, inhibited the active transport of glucose by glucose transporter GLUT2 translocation in both directions. Therefore, the intensity of fluorescence for cells treated with phloretin was less visible under the microscope. This indicates the lower number/density of GLUT2 receptors on the cell surface. On the other hand, glucose present (4.5 g/L) in the culture medium is responsible for the translocation of some GLUT2 receptors. Further increase in glucose concentration led to significantly increased GLUT2 translocation. Complete elimination of glucose from the medium is impossible due to the inhibition of cell proliferation and induction of apoptosis. In experiments with a medium containing a glucose concentration of 1 g/L, a significant decrease in the viability of Caco-2 cells was observed. Lowered glucose content in the culture medium may result in the translocation of GLUT2 receptors. The HR extract at concentrations of 50 and 100 μg/ml (in conditions of increased glucose 75 mM) shows a similar expression of GLUT2 receptors to the untreated control (4.5 g/l glucose) and control cells grown in a low-glucose environment (1 g/l). In the concentration range of 500–2000 μg/ml, an increased expression of GLUT2 receptors was observed, similar to the expression observed in control cells in the environment of increased glucose concentration (75 mM).

### 3.6 Phytochemical analysis

**FIGURE 6:**
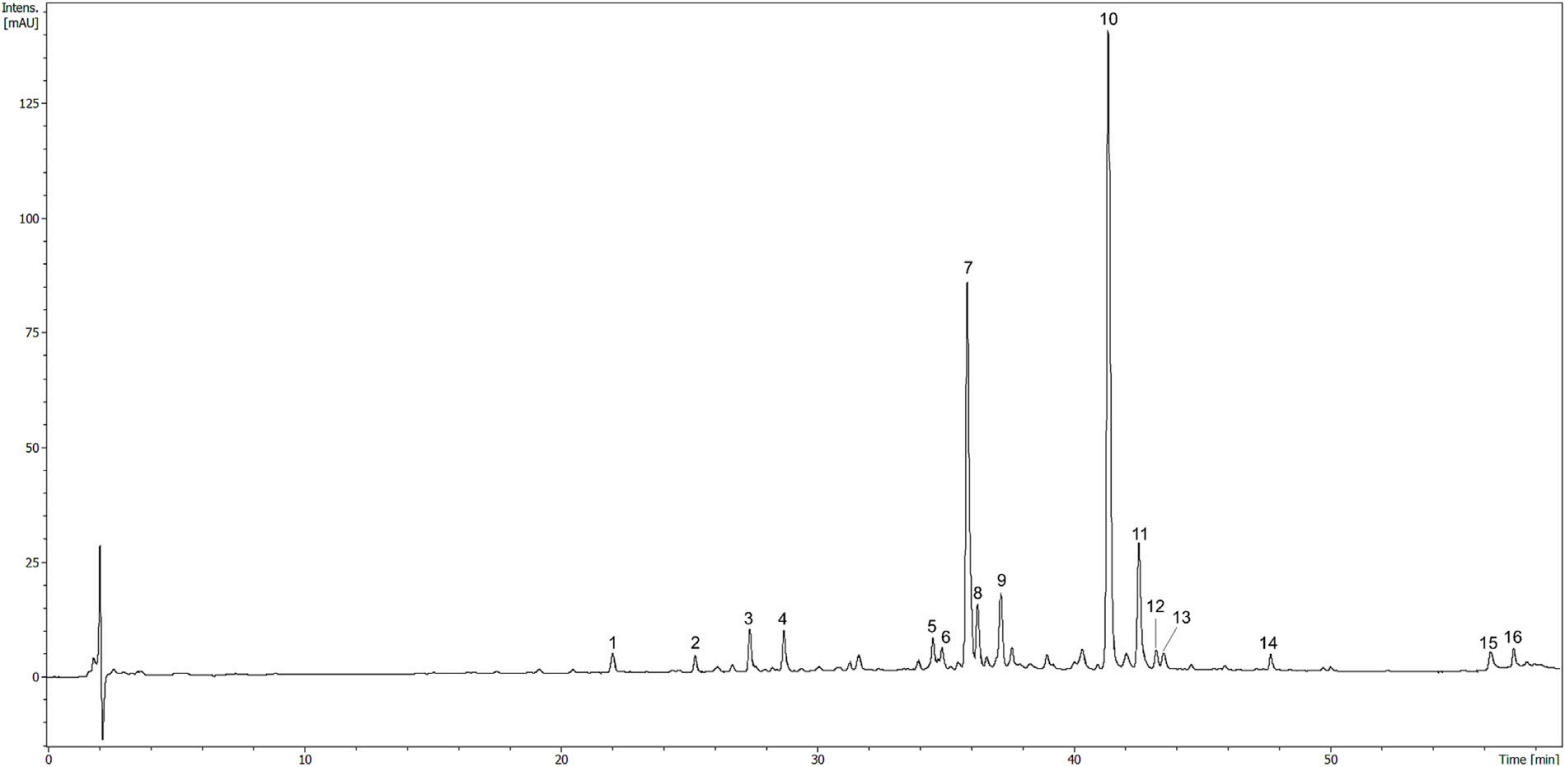
HPLC chromatogram of HR (10 mg/ml) registered at 325 nm. The number codes were provided in Table 1.

Total polyphenol content in the HR extract was 33.0 ± 3.6 µg GAE/mg of the dry extract. The major compounds identified in the HR extract included glycosides of isorhamnetin, as well as glycosides of quercetin and kaempferol to a lesser extent (Table 1). The compounds were assigned according to their MS data and available literature (Fang et al., 2013; Siegień et al., 2021). Three glycosides of isorhamnetin were isolated from the methanolic fraction of HR as it was described in the Supplementary Material. The most abundant constituents of HR extract (Figure 6) were compounds assigned to isorhamnetin-3-*O*-*β*-D-glucosyl-7-*O*-*α*-L-rhamnoside (7, Rt = 36.1 min) and isorhamnetin-3-*O*-*α*-L-rhamnosyl-(1→6)-*β*-D-glucoside (10, Rt = 41.5 min) (Fang et al., 2013). The main ion in the MS spectrum of these compounds was [M-H]^-^ *m*/*z* 623. The major MS^2^ ion in a negative ionization mode was [isorhamnetin-H]^-^ *m*/*z* 315.

**TABLE 1:** Data of mass spectrometry for compounds tentatively assigned in HR.

No	Compound	Retention Time [min]	*λ*_max_ [nm]	[M-H]^-^ *m/z*	MS/MS
1	Isorhamnetin glycoside*	22.0	280, 330	931	913, 813, 769, 423, 315
2	Quercetin glycoside*	25.6	270, 360	771	625, 446, 301
3	Kaempferol glycoside*	27.6	265, 344	755	623, 609, 285
4	Isorhamnetin glycoside*	28.9	265, 354	785	639, 607, 459, 315
5	Isorhamnetin glycoside*	34.5	265, 355	769	737, 605, 503, 423, 315
6	Kaempferol glycoside*	35.3	270	593	447, 431, 285
7	**Isorhamnetin 3-*O*-*β*-D-Glc-7-*O*-*α*-L-Rha**	36.1	250, 270, 354	623	477, 315
8	Isorhamnetin glycoside*	36.5	270, 338	769	623, 461, 315
9	Isorhamnetin feruloyl-glycoside*	37.4	255, 351	961	816, 639, 315
10	**Isorhamnetin-3-*O*-*α*-L-Rha-(1→6)-*β*-D-Glc**	41.5	254, 354	623	623, 477, 315
11	**Isorhamnetin 3*-O*-*β*-D-Glc**	42.7	255, 354	477	314, 285
12	Isorhamnetin derivative	43.4	265, 354	593	477, 315
13	Isorhamnetin malyl-glycoside*	43.8	254, 354	739	623, 315
14	Isorhamnetin derivative	47.9	270, 344	637	965, 623, 482, 341, 315
15	Isorhamnetin derivative	56.5	270, 369	461	446, 315
16	Kaempferol glycoside*	57.4	270, 350	593	447, 285

Glc, glucosyl/glucoside; Rha, rhamnosyl/rhamnoside; in bold—isolated compounds; * assigned based on the available literature (Fang et al., 2013).

## 4 Discussion

The ethnomedicinal use of sea buckthorn has been widely known in the Eurasian regions. Apart from external use in wound healing, psoriasis, and atopic dermatitis (Pundir et al., 2021), preparations from fruits are particularly recommended in everyday diet for internal application (Olas and Skalski, 2022). Many indications were reported for sea buckthorn preparations due to their beneficial influence on hemostasis, e.g, within cardiovascular diseases (Olas and Skalski, 2022). In the traditional Chinese medicine its fruits were used in the treatment of hepatic disorders, fever, cold, toxicity, inflammation, metabolic disorders, cough and gynecological diseases (Olas, 2018). There is evidence that systemic low-grade inflammation caused by increase of LPS concentration leads to the development of a wide range of chronic disorders (Mohammad and Thiemermann, 2020). Bearing in mind the traditional use of sea buckthorn, in the present study, the potential effect of extract from the fruit of sea buckthorn, which is a well-known, health-beneficial nutrient used in everyday diet, on metabolic-related low-grade inflammation was evaluated. The main goal of the study was an evaluation of the role of flavonoids detected in HR extract in the cross-talk between the intestinal epithelium and immune cells. Taking into consideration the influence of LPS leakage through intestinal epithelium in the low-grade inflammation in chronic disorders, we established for the first time that HR extract rich in isorhamnetin glycosides at the lowest tested concentration decreased LPS concentration on the basolateral side of the Caco-2 monolayer. In this model, it also significantly inhibited the secretion of chemotactic cytokine such as IL-8, which is responsible for directional movement of leukocytes. Since epithelial cells may be affected by higher concentrations of phytochemicals in the intestine, we decided to test the effect of the extract in the concentration range from 50 to 2000 μg/ml on Caco-2 cells. However, considering the limitations in the bio-accessibility of phytochemicals from plant extracts, we decided to use the decreasing concentration system and the lower concentrations of HR extract were tested for the immune cells such as PMN and PBMC. Additionally, bearing in mind that hyperglucose is one of the factor initiating the dysfunctions of immune cells in the disease conditions, we considered the effect of the extract on glucose absorption *via* the GLUT2 receptor.

A complex network of immune cells in the deep part of the gut barrier contains up to 70% of the body’s immune cells. Therefore, in our study we used two models of immune cells, including PMN and PBMC, which actively secrete cytokines after stimulation with LPS from *E. coli*. Granulocytes are the first cells migrating into tissue sites as part of the host defense system. In particular, they are the most abundant cell type in intestinal lesions in inflammatory bowel disease (IBD) (Nikolaus et al., 1998). Human peripheral blood mononuclear cells include lymphocytes (T cells, B cells, and NK cells; 70–90%), monocytes (10–20%), and dendritic cells (1–2%). These immune cells are the main players in the cross-talk between intestinal cells and immune system. They are involved in the maintenance of the intestinal homeostasis. This cellular model is often used for a determination of anti-inflammatory activity of plant materials (Leelawat and Leelawat, 2018). It provides increased variance due to physiological changes between donors. It is well known that a wide range of physiological factors, such as nutritional status, hormone levels and infections/inflammation, influence the reactivity of immune cells. Therefore, the usage of *in vivo*-related models of PMN and PBMC from several donors in our study enhances the strength of reproducing results and supports their generality. Lipopolysaccharide mainly induces B cell proliferation and activation of monocytes [35]. The main function of immune cells is to protect against pathogenic microorganisms and maintain immune tolerance to commensal bacteria (Lopetuso et al., 2015). In addition, the integrity of the structures within the gut barrier is necessary for the maintenance of normal intestinal permeability. Any alteration of the intestinal equilibrium leads to the passage of the luminal content to tissues, and into the bloodstream (Wells et al., 2017). This phenomenon, known as gut barrier leakage, results in the excessive activation of the immune response, both within the intestinal epithelium and systemic (Lopetuso et al., 2015). It is widely considered that gut barrier leakage is an etiopathogenetic factor for the development of plenty of diseases such as infectious enterocolitis, IBD, irritable bowel disease, small intestinal bacterial overgrowth (SIBO), food intolerance, and atopic symptoms (Bischoff et al., 2014). The role of gut microbiota has been recently indicated as the major factor responsible for a condition of the intestinal barrier. The concentration of bacteria adhering to the enterocytes is often higher in intestine-related disorders as well as it correlates with the pathological severity. In particular, changes in bacterial diversity, such as an increase in Enterobacteriaceae, including *Escherichia coli*, and *Bacteroidetes*, are engaged in the inflammation involving disorders like IBD, obesity, and celiac disease. Lipopolysaccharide from Enterobacteriaceae exacerbates intestinal injuries*.* In addition, an increase in both *Firmicutes* and *Proteobacteria* is particularly observed in gastrointestinal disorders (Murphy et al., 2015; Zeng et al., 2017). The pathogenic bacteria particularly adhere to intestinal epithelial cells and break the integrity of enterocytes wall. These alterations lead to weakening the intercellular adhesion and inflammatory response. Several inflammatory cytokines including IFN-*γ*, TNF-*α*, IL-1*β*, and IL-17 have been known to cause an increase in intestinal permeability (Wells et al., 2017). Therefore, the pharmacological modulation of the intestinal barrier is the key factor in clinical practice. At present, corticosteroids, aminosalicylates like mesalazine (5-aminosalicylic acid, 5-ASA), and biological medicines like anti-TNF-*α* are mainly used in gastroenterology therapies ((CHMP), 2018a; b).

Interestingly, a high-fat diet (HFD) significantly increases the serum level of LPS, which is similar to a continuous infusion of LPS (Cani et al., 2007). It is known that HFD-induced alterations in gut microbiota are associated with obesity and low-grade chronic inflammation (Murphy et al., 2015). On the other hand, plant-derived secondary metabolites such as polyphenols, which are widely distributed in many natural products, are supposed to be involved in stimulating the growth of beneficial bacteria such as *Lactobacillus* spp. and *Bifidobacterium* spp. (Corrêa et al., 2019). Therefore, the regulation of the composition of gut microbiota with a diet seems to be crucial for intestinal epithelium (Kumar Singh et al., 2019). Based on the 16S rRNA gene sequencing analysis, recent reports showed that sea buckthorn freeze-dried powder or high-flavonoid extract had improved the composition of gut bacteria. The preparations lowered the ratio of *Firmicutes*/*Bacteroidetes* and increased a relative abundance of *Akkermansia*. These changes revealed a positive correlation with lipolysis genes and negatively correlated with lipid synthesis (Guo et al., 2020; Zhao et al., 2022). Moreover, the HR fruit extracts showed positive effects in cisplatin-induced jejunum mucositis in rats. It was demonstrated that HR extract reversed the biochemical changes induced by cisplatin such as the increase of malondialdehyde and proinflammatory cytokines, including IL-1*β* and TNF-*α*, as well as a decrease of oxidative parameters. It was concluded that extract of sea buckthorn fruit might exert a protective effect on the oxidative damage of intestinal tissue (Arslan et al., 2018). Furthermore, the beverages from the fruits of sea buckthorn were used to support the supplementation of *Lactobacillus rhamnosus* GG in the attenuation of LPS-induced colonic tissue damage in zebrafish (Sireswar et al., 2020). Additionally, the recent report concerning polysaccharide fraction from berries of sea buckthorn indicated these classes of compounds as responsible for a positive effect on body weight, caloric intake, and average cell size of white and brown adipocytes tissue (Ma Z. et al., 2022).

Moreover, polyphenols are believed to show an anti-allergic effect through pathways and immune cell functions engaged in the allergic immune response. Flavonols, such as quercetin, and flavan-3-ols like (epi)catechin, as well as anthocyanidins, procyanidins, and resveratrol, are the most investigated polyphenols. Their intake is likely to assure the balance of T-helper (Th) type 1 and 2 cells, suppress IL-4 and IL-13 synthesis (Th2 type cytokines) by effector cells (basophils or mast cells) stimulated with allergen or anti-IgE antibody, or even suppress antigen-specific IgE antibody formation (Mlcek et al., 2016). In general, allergies are considered autoimmune disorders resulting from the accelerated response of the autoimmune system against some antigens. Some bacteria-derived metabolites contribute to immune maturation and development. Dysbiosis has been implicated in the pathogenesis of immune-mediated disorders, such as allergic diseases. The regulation of gut microbiota by the stimulation of beneficial bacterial strains is one mode of therapeutical approach although the precise effects of the microbiome on the pathogenesis of allergic diseases remain unknown (Han et al., 2021). Apart from the prevention of dysbiosis, physical protection of the gut barrier against LPS leakage is hypothesized to be a crucial and direct approach. In the recent report three dietary patterns, including “high-meat” (meat and sweetened drink), “prudent diet” (fish, fruit, legumes, and vegetables), and “high alcohol” (higher alcohol consumption) were recognized as important for the state of the gut microbiome. Their influence on circulating cytokines through the gut microbiome, as a mediator between diet and immune system, was evaluated (Shi et al., 2022). Food and plant-derived products are the sources of compounds, which directly affect the intestinal epithelium in addition to the gut microbiome. The key question is which compounds are mainly responsible for the health-beneficial effect of a diet and why. Therefore, we tested the influence of HR extract rich in flavonoids, such as isorhamnetin derivatives, on the permeability of LPS through a Caco-2 monolayer *in vitro*.

To study LPS efflux and GLUT2 translocation, we used dedicated higher concentrations of HR extract assuming that higher concentrations of plant extracts might directly affect the intestinal epithelium. In our study, the detected amounts of LPS in the case of samples treated with HR extract at the concentrations of 1000 and 2000 μg/ml were similar to that of LPS-treated control. Therefore, we hypothesized that some non-specific reactions of polysaccharides from HR extract (Wang et al., 2018) may take place in the assay used in this study. However, despite the HR itself did not affect the viability of Caco-2 cells, the cytotoxic effect of LPS potentiated by HR might be taken into consideration. Lipopolysaccharides of Gram-negative bacteria contain a biphosphorylated lipid, named lipid A, and a hydrophilic polysaccharide. Lipid A forms the matrix of the outer membrane appendix, which is stabilized by divalent cations. Polysaccharide moiety extends outward from the bacteria. It consists of an oligosaccharide core, containing 10-12 sugars, and the *O*-specific polysaccharide chain. The core of LPS molecules is covalently bound through acidic sugar, usually 3-deoxy-D-manno-oct-2-ulopyranosonic acid, to the lipid A (Nalbantsoy et al., 2011). Both plasma endotoxin (300 pg/ml) and lipopolysaccharide-binding protein (31.2 μg/ml) levels were elevated in the patients suffering from severe sepsis and/or septic shock (Opal et al., 1999). Endotoxins have a variety of effects on cell cultures. The effects of interactions between endotoxins and membranes, such as morphological changes like surface ruffles and increased organelles, large vacuoles in the cytoplasm, or morphological damage, vary significantly for different cell types and cell lines (Nalbantsoy et al., 2011). In general, membranes are critical for the synthesis of cell products and endotoxins can influence these processes. In our study, we used LPS at the concentration of 100 ng/ml, whereas in most other reports the toxic effect of LPS on cells was induced by LPS at the concentration of 1 μg/ml or even higher (Yamamoto et al., 1994; Peluso et al., 2010; Nishio et al., 2013; Serreli et al., 2019). However, we suppose that the phytochemicals of HR extract in higher concentrations may increase the cytotoxicity of LPS for cells leading to partial damage of the monolayer enabling LPS leakage. For this reason, we probably did not observe a significant decrease in LPS concentration on the basolateral side when the Caco-2 monolayer was treated with HR extract at the concentrations of 1000 and 2000 μg/ml.

Lipid A determines major biological activities, including the toxicity of LPS. It was established that detoxified, lipid-free LPS had no cytotoxic effect on cells like macrophages (Yamamoto et al., 1994). Lipopolysaccharide was indicated as a potent inducer of NO production although the LPS-triggered NO production in macrophages requires an additional signal of IFN-*γ*. Low molecular weight DNA fragments were found in lysates of IFN-*γ*/LPS treated macrophages, which were characterized by a low rate of viability (Yamamoto et al., 1994). In the intestinal epithelium, NO production is a result of activation of the inducible nitric oxide synthase (iNOS). Chronic release of NO at high concentrations, caused by up-regulation of iNOS, has been correlated with the pathogenesis of intestinal disorders like IBD or colon cancer (Serreli et al., 2019). The cumulation of peroxynitrite, formed in the environment of oxidative stress, can lead to the loss of cell function or cell death. Thus, an excessive and prolonged iNOS-derived NO production can lead to injuries of enterocytes, an increase of mucosal permeability like in the case of IBD (Serreli et al., 2019). Activation of the mitogen-activated protein kinases (MAPK) pathways, including extracellular signal-regulated kinases (ERK1/2), c-Jun N-terminal kinases (JNK), and p38 MAPK, is typical for oxidant-induced apoptosis due to the targeting of nuclear factor κ-light-chain-enhancer of activated B cells (NF-κB) by pro-inflammatory agents (Ryter et al., 2007). Lipopolysaccharide can induce the phosphorylation of protein kinase B (Akt), IκB, as well as p38 MAPK, ERK1/2, and JNK. Therefore, inhibition of tyrosine kinases seems to be a target for the prevention of the damages caused by LPS. A tyrosine kinase inhibitory activity of flavonoids, such as quercetin, genistein, myricetin, and others, as well as the attenuation of LPS-induced cytotoxicity in the bovine endothelial cells were reported (Melzig and Loose, 1998). Some simple phenols, such as hydroxytyrosol and tyrosol, have been already known for counteracting of too excessive iNOS expression by the down-regulation of the kinases resulting in inhibition of NO over-release in Caco-2 cells and prevention of oxidative injuries of enterocytes (Serreli et al., 2019). Other antioxidants such as tocopherols and tocotrienols, which occur in the fruit pulp or oil of sea buckthorn (Pundir et al., 2021), are incorporated into the cultured cells and suppressed LPS cytotoxicity (Nishio et al., 2013). Taking into consideration this evidence, it is likely that constituents of HR extract at higher concentrations may not prevent LPS leakage due to the increased cytotoxicity of LPS in Caco-2 cells. Further investigation, including iNOS expression and cytotoxicity of HR co-treatment with LPS, is required to fully elucidate this phenomenon.

On the other hand, the HR extract in a lower concentration of 500 μg/ml significantly decreased LPS concentration on the basolateral side. For this reason, we hypothesize that the plant extracts rich in flavonoids regularly provided with a diet even at lower concentrations may prevent LPS leakage through the intestinal epithelium. Considering this effect, one of the proposed modes of action is the binding of LPS by flavonoids, which protect epithelial cells against its effects. It must be underlined that in the empirical experiments we observed that the fractions characterized by the high content of flavonoids cover the wells with a usually yellow and lipophilic layer. Indeed, the solubility of flavonoids in polar solvents is limited as it was in the case of the compounds 7, 10, and 11 (Supplementary Material). We presume that the plant-extracts rich in polyphenols, particularly flavonoids, may physically protect the gut barrier *in vivo*. Nevertheless, the functionality of intestinal epithelial barriers depends mainly on the intercellular junctions. Tight junctions (TJ) are composed of transmembrane proteins such as claudins, occludin, junctional adhesion molecule, and zonula occludens proteins. They are regulators of paracellular permeability (Barbara et al., 2021). While the paracellular cross of water and electrolytes is physiologically desired for osmosis and cell polarity, the alterations of the intestinal epithelial barrier open the way for excessive passage of dietary derived molecules and PAMPs, including LPS. That induces immune activation involved in the pathogenesis of the intestinal and systemic disease such as diabetes, obesity, non-alcoholic liver diseases (Barbara et al., 2021). It should be underlined that cytokines secreted by immune cells are also engaged in the increase of intestinal epithelial TJ permeability (Al-Sadi and Ma, 2007). We hypothesize that phytochemicals of HR extract may influence the expression of TJ, which requires further investigation.

The activity stabilizing membranes may prevent the formation of the complex binding LPS with its targeting proteins like pattern recognition receptors (PRR), e.g, TLR4. Thus, the inflammatory signal is not propagated. It is worth noting that heterodimerization of TLR4 with myeloid differentiation protein-2 (MD-2) is required for the cell surface localization of TLR4, the activation of TLR4 by LPS, as well as further responsiveness to LPS. It was reported that curcumin and prenylated chalcone-type flavonoids represented by xanthohumol are capable to suppress the LPS-induced inflammatory signaling through direct competitive binding to the hydrophobic MD-2 pocket (Peluso et al., 2010). Isorhamnetin showed a high binding affinity to hydrophobic pocket of MD-2, which is the site of LPS-binding. This interaction was described by the association rate of 2.296 × 10^2^/(M × s) and dissociation rate of 1.242 × 10^−3^/s (Chauhan et al., 2019). Even though isorhamnetin derivatives prevent LPS-binding with TLR4 *via* blocking MD-2, the prevention of LPS leakage might be an additional systemic benefit of their activity. That could also probably explain their inhibition of IL-8 secretion by Caco-2 cells in our research.

It was previously shown that mitochondrial dysfunction and oxidative stress caused by the non-steroidal anti-inflammatory drugs (NSAIDs) may lead to the alteration in the distribution of certain tight junction proteins, resulting in an increase in paracellular permeability. Quercetin itself as well as onion peel aqueous extract rich in quercetin metabolite were proven to protect Caco-2 monolayers against oxidative stress and an increased permeability induced by NSAIDs (Fuentes et al., 2021). It is worth noting that products containing onion extract, a rich source of quercetin, have been used externally to treat scars to relieve allergic symptoms after itching (Teshika et al., 2019). The structure of isorhamnetin differs from the structure of quercetin by the presence of the *O*-methoxy group instead of the *O*-hydroxyl group in C-3′. Although isorhamnetin is a less effective antioxidant than its unmethylated precursor, it is capable to inhibit LPS-induced production of reactive oxygen species (ROS), preventing cell death and inflammatory response *in vivo*. Isorhamnetin is characterized by higher metabolic stability and better intestinal absorption (Seo et al., 2014). Methylation increases the hydrophobicity of flavonoids, which significantly influences their biological activity (Wen et al., 2017). Thus, the HR extract containing a wide range of isorhamnetin glycosides seems to be a promising natural product. Therefore, we attempted to answer the question if flavonoids may exert their biological effect in any different mode of action that direct influence immune cells. The synthesis of cytokines is induced by many bacterial components such as cell surface polysaccharides, protein A, peptidoglycans, lipid A-associated proteins, or lipoproteins. Many cells can express TLRs, which are also found in Caco-2. Furthermore, bacteria-derived products may damage cellular membranes leading to the leakage of some molecules (Hosoi et al., 2003). The inflammatory effect of LPS is generated mainly by TLR4 signaling. Extracellular proteins, such as LPS-binding protein, CD14, and G_i_ protein participate in transferring of LPS to a signaling complex composed of MD2 and MyD88 or mediating LPS effects. The stimulation of immune cells by LPS activates MAPKs, which regulate gene expression through phosphorylation of intracellular proteins and transcription factors (Tucureanu et al., 2018). Cytokines are key mediators in response to infection. They are translocated across the endoplasmic reticulum through the Golgi apparatus to the plasma membrane (Duitman et al., 2011). Our results suggest that HR can prevent both LPS leakage and the effects resulting from activation of TLRs, including cytokine secretion by leukocytes.

Reactive oxygen species are known for their role in the regulation of metabolic and inflammatory diseases. Cytoplasmic ROS production by the NOX family of enzymes is mainly responsible for disease pathophysiology. In general, ROS promote the activation of NF-κB, resulting in the production of pro-inflammatory mediators and cytokines. Inflammatory agonists like IL-1*β* induce endosomal IL-1 complex, which involves MyD88 and NADPH oxidase 2 (NOX2) in the endosomal recruitment of TNF receptor 6. On the other hand, NOX4 is necessary for LPS-induced NF-κB activation. Furthermore, NF-κB activation by TNF-*α* increases antioxidant expression and decreases apoptotic signaling (Forrester et al., 2018). There is no doubt that both ROS and inflammatory agents participate in the interdependent, inflammatory pathways. For this reason, their reduction is necessary for cellular and tissue homeostasis. The antioxidant properties of sea buckthorn berries as well as isorhamnetin were previously described (Kim et al., 2019; Tkacz et al., 2019). In our study, we assessed TPC, which was 33.0 ± 3.6 µg GAE/mg of the dry mass of extract (3.3 g/100 g). According to the previous report, the content of phenols in the dry mass of fresh sea buckthorn berries was ranged from 468.60 to 901.11 mg/100 g (Tkacz et al., 2019). Taking into consideration a relevant content of phenolic compounds in our tested extract, we can suppose that phenols of HR due to their known antioxidant properties might participate in the reduction of cytokine secretion. The potential mode of this action might be through the inhibition of NF-κB pathway. On the other hand, the nuclear factor erythroid 2-related factor 2 (Nrf2) is a transcription factor which responds to oxidative stress. It binds to the antioxidant response element (ARE) in the genes coding antioxidant enzymes like NAD(P)H:quinone oxidoreductase one and proteins for glutathione synthesis (Vomhof-Dekrey et al., 2012). The nuclear factor 2 is constitutively degraded through binding to Kelch-like ECH-associated protein 1 (Keap1) while ROS inhibit ubiquitination of Nrf2 and stimulate its cumulation. The upregulation of heme oxygenase-1 (HO-1) gene inhibits proinflammatory cytokines and activates anti-inflammatory cytokines. Some phytochemicals are able to activate Nrf2 (Xu et al., 2022). It seems that the regulatory functions of Nrf2 pathway, e. g. in adipocyte differentiation and liver metabolism, might be affected by constituents of HR extract. The activation of Nrf2, which increases energy metabolism and conversely suppresses lipid synthesis, seems to be a potential mechanism of HR activity.

We believe that the prevention of LPS leakage, inhibition of TLRs activation, inhibition of phosphorylation pathways of MAPKs or activation of Nrf2/ARE pathway by HR potentially participate in the regulation of inflammasome formation and the cross-talk between the epithelial and immune cells, in particular when the gut barrier is damaged like in chronic bowel diseases. Moreover, chronic low-grade inflammation has long been associated with the promotion of type 1 diabetes but is even more often responsible for the onset and/or development of type 2 diabetes. Therefore, we attempted to establish the effect of the HR extract on intercellular cross-talk between epithelial and immune cells. We believe that inhibition of IL-8 by the HR extract in the Caco-2 cells is likely to prevent the too excessive and prolonged response of immune cells to chemoattractant, which supports the resolution of inflammation within the epithelium. Further inhibition of TNF-*α* release by PMN and PBMC stays in agreement with anti-TNF-*α* therapies used in clinical practice of chronic intestinal disorders. On the other hand, the HR extract increased the release of IL-1*β* by PMN and did not influence IL-6 secretion by PBMC. The isolated isorhamnetin glycosides, such as compounds 7, 10, and 11, also increased the secretion of IL-1*β* (Supplementary Figure S14). In addition, we suppose that some other phytochemicals of HR extract, such as polysaccharides, may show an immune-inducing effect. Polysaccharides are known for their immune-stimulating activity through the induction of cytokine secretion (Deng et al., 2018). The screening of cytokine secretion by isorhamnetin glycosides in the PMN model revealed that the isolated flavonoids themselves did not inhibit significantly secretion of TNF-*α*, except for compound 7 (25 µM), and IL-8 (Supplementary Figure S14). Therefore, we suppose that the synergistic or additive effects of phytochemicals take place in the case of HR extract. The results of studies conducted in a model of alcohol-induced liver injury (AFLD) showed that the levels of selected cytokines such as TNF-*α*, TGF-*β*, and IL-6 were reduced after treatment of mice with high-flavonoid extract of HR. The background for this activity seems to be the decreased protein and mRNA expression of p65 NF-κB and p38 MAPK in the liver of mice with AFLD (Zhao et al., 2022). Apart from diminished expressions of NF-κB, ERK, and p38, total flavonoids of HR also decreased concentrations of IL-1*β* and IL-6 released by HaCaT cells treated by IFN-*γ*/TNF-*α in vitro* (Gu et al., 2022). We confirmed that Caco-2 cells had secreted IL-8 mainly in response to IL-1*β* stimulation (Eckmann et al., 1993). However, HR extract seems to be able to decrease IL-8 secretion even in the conditions of augmented concentrations of IL-1*β*. It was previously established that isorhamnetin isolated from the fruit of sea buckthorn inhibited the IL-1*β*-stimulated synthesis of NO and prostaglandin E2, the expression of NF-κB and transcription factor p65, as well as the degradation of NF-κB inhibitor, iNOS, and prostaglandin G/H synthase two in chondrocytes (Li et al., 2017). In addition, it down-regulated cytokines and inhibited the expression of JNK, p38, and NFκB proteins in the mouse brain, alleviating neuroinflammation caused by high-fat diet (Mulati et al., 2021). On the other hand, isorhamnetin regulated Nrf2/Keap1 pathway enhancing the expression of Nrf2, HO-1, superoxide dismutase (SOD)1 and SOD2 in cigarette-induced chronic obstructive pulmonary disease in mice (Xu et al., 2022). Taken together, we proved that HR extract rich in isorhamnetin derivatives reduced levels of pro-inflammatory cytokines at different steps of developing chronic low-grade inflammation in addition to an increase of an anti-inflammatory cytokine such as IL-10. We suppose that the inhibition of pro-inflammatory cytokines secretion by HR extracts in our study might be explained by the inhibition of MAPKs and NF-κB signaling or activation of Nrf2 signaling pathways.

According to our knowledge, there is no data concerning the potential effect of HR on glucose transport, which takes place in the intestine. In our previous study, we established IC_50_ values of HR extract for pancreatic lipase (59.7 ± 4.6 μg/ml) and *α*-amylase (83.0 ± 7.8 μg/ml) (Siegień et al., 2021). We suppose that isorhamnetin glycosides significantly participate in this activity. It was found that oral administration of isorhamnetin for 14 weeks significantly reduced the body weight, food intake, liver lipid level, and serum lipid level of high-fat and high fructose diet-fed mice (Mulati et al., 2021). Therefore, we decided to search for a further potential effect of HR extract on selected glucose transporter such as GLUT2. This transporter is present in the enterocyte apical area. After sugar absorption even at low doses (<10 mM) it is colocalized with brush border actin in the upper half of the intestinal villus (Zheng et al., 2012; Grefner et al., 2015). Translocation of cytosolic GLUT2 from cytosolic vesicles into the apical membrane enables the capacity of enterocytes for transcellular glucose uptake (Zheng et al., 2012). In our study, HR extract in lower studied concentrations diminished the fluorescence of GLUT2 similarly to the non-treated control. It was previously established that flavonols, such as quercetin, quercetin 3-*O*-glucoside (isoquercitrin), and myricetin, inhibited 2-deoxyglucose and fructose uptake in Caco-2 cells. It is highly possible that some flavonoids are transported into enterocytes and they can inhibit basolateral GLUT2 transport, and intestinal glucose and fructose absorption (Kwon et al., 2007). Thus, it is possible that inhibition of GLUT2 translocation by HR extract makes it a potential plant preparation preventing diabetes mellitus and other disorders included in metabolic syndrome.

## 5 Conclusion

There is evidence that suggests the role of medicinal plant, such as sea buckthorn, in modulating both acute and chronic inflammation providing the background for the alleviation of inflammation-linked metabolic disorders like diabetes mellitus and obesity. In the present study, we established that flavonoids found in the extracts of sea buckthorn fruit directly prevent leakage of LPS through the intestinal barrier, which may further protect against the development of low-grade inflammation. The ability of the studied extract to inhibit the cytokine secretion both by epithelial and immune cells may additionally potentiate the resolution of inflammation accompanying the metabolic disorders. Despite the effect of *H. rhamnoides* fruit preparations on the gut microbiome and its enhancement being well-known, the direct protection of intestinal epithelium by polyphenols(flavonoid)-rich extract has never been considered. The additional capability of sea buckthorn preparation for inhibition of glucose transport by inhibiting GLUT2 translocation in the enterocytes justifies the necessity of the regular uptake of polyphenols/flavonoids in a diet even at low concentrations to maintain metabolic and immune homeostasis. The prevention of intestinal barrier dysfunction can be exploited to improve health using dietary therapeutics such as preparations of sea buckthorn containing mainly isorhamnetin glycosides. We believe that the results of this study might support the pleiotropic significance of sea buckthorn in the traditional medicine.

## Data Availability

The original contributions presented in the study are included in the article/Supplementary Material, further inquiries can be directed to the corresponding author.

## References

[B1] Al-Sadi R. M. Ma T. Y. (2007). IL-1β causes an increase in intestinal epithelial tight junction permeability. J. Immunol. 178 (7), 4641–4649. 10.4049/jimmunol.178.7.4641 17372023 PMC3724221

[B2] Arslan A. Ozcicek F. Cimen F. K. Nalkiran H. S. Gulaboglu M. Cetin N. (2018). Effects of *Hippophae rhamnoides* extract on oxidative mucosal injury induced by cisplatin in rat jejunum. biomedicalresearch 29, 401–407. 10.4066/biomedicalresearch.29-17-2913

[B3] Barbara G. Barbaro M. R. Fuschi D. Palombo M. Falangone F. Cremon C. (2021). Inflammatory and microbiota-related regulation of the intestinal epithelial barrier. Front. Nutr. 8, 718356. 10.3389/fnut.2021.718356 34589512 PMC8475765

[B4] Bischoff S. C. Barbara G. Buurman W. Ockhuizen T. Schulzke J. D. Serino M. (2014). Intestinal permeability--a new target for disease prevention and therapy. BMC Gastroenterol. 14, 189. 10.1186/s12876-014-0189-7 25407511 PMC4253991

[B5] Böyum A. (1968). A one-stage procedure for isolation of granulocytes and lymphocytes from human blood. General sedimentation properties of white blood cells in a 1g gravity field. Scand. J. Clin. Laboratory Investigation Suppl. 97, 51–76. 4179067

[B6] Cani P. D. Amar J. Iglesias M. A. Poggi M. Knauf C. Bastelica D. (2007). Metabolic endotoxemia initiates obesity and insulin resistance. Diabetes 56 (7), 1761–1772. 10.2337/db06-1491 17456850

[B7] Chauhan A. K. Kim J. Lee Y. Balasubramanian P. K. Kim Y. (2019). Isorhamnetin has potential for the treatment of *Escherichia coli*-induced sepsis. Molecules 24 (21), 3984. 10.3390/molecules24213984 PMC6864442 31689976

[B8] Ciesarová Z. Murkovic M. Cejpek K. Kreps F. Tobolková B. Koplík R. (2020). Why is sea buckthorn (*Hippophae rhamnoides* L.) so exceptional? A review. Food Res. Int. 133, 109170. 10.1016/j.foodres.2020.109170 32466930

[B9] Cohen M. Kitsberg D. Tsytkin S. Shulman M. Aroeti B. Nahmias Y. (2014). Live imaging of GLUT2 glucose-dependent trafficking and its inhibition in polarized epithelial cysts. Open Biol. 4, 140091. 10.1098/rsob.140091 25056286 PMC4118605

[B10] Committee for Medicinal Products for Human Use (CHMP) (2018a). Guideline on the development of new medicinal products for the treatment of Crohn’s disease. London: European Medicine Agency.

[B11] Committee for Medicinal Products for Human Use (CHMP) (2018b). Guideline on the development of new medicinal products for the treatment of ulcerative colitis. London: European Medicine Agency.

[B12] Corrêa T. A. F. Rogero M. M. Hassimotto N. M. A. Lajolo F. M. (2019). The two-way polyphenols-microbiota interactions and their effects on obesity and related metabolic diseases. Front. Nutr. 6, 188. 10.3389/fnut.2019.00188 31921881 PMC6933685

[B13] Czerwińska M. E. Bobińska A. Cichocka K. Buchholz T. Woliński K. Melzig M. F. (2021). *Cornus mas* and *Cornus officinalis*-A comparison of antioxidant and immunomodulatory activities of standardized fruit extracts in human neutrophils and caco-2 models. Plants (Basel) 10 (11), 2347. 10.3390/plants10112347 34834710 PMC8618406

[B14] Czerwińska M. E. Duszak K. Parzonko A. Kiss A. K. (2016). Chemical composition and UVA-protecting activity of extracts from *Ligustrum vulgare* and *Olea europaea* leaves. Acta Biol. cracov. Bot. 58 (2), 45–55. 10.1515/abcsb-2016-0016

[B15] Deng X. Liu Q. Fu Y. Luo X. Hu M. Ma F. (2018). Effects of *Lycium barbarum* polysaccharides with different molecular weights on function of RAW264.7 macrophages. Food Agric. Immunol. 29, 808–820. 10.1080/09540105.2018.1457628

[B16] Desai M. S. Seekatz A. M. Koropatkin N. M. Kamada N. Hickey C. A. Wolter M. (2016). A dietary fiber-deprived gut microbiota degrades the colonic mucus barrier and enhances pathogen susceptibility. Cell 167 (5), 1339–e21. 10.1016/j.cell.2016.10.043 27863247 PMC5131798

[B17] Duan J. Dang Y. Meng H. Wang H. Ma P. Li G. (2016). A comparison of the pharmacokinetics of three different preparations of total flavones of Hippophae rhamnoides in beagle dogs after oral administration. Eur. J. Drug Metab. Pharmacokinet. 41 (3), 239–249. 10.1007/s13318-015-0254-9 25613316

[B18] Duitman E. H. Orinska Z. Bulfone-Paus S. (2011). Mechanisms of cytokine secretion: A portfolio of distinct pathways allows flexibility in cytokine activity. Eur. J. Cell Biol. 90 (6-7), 476–483. 10.1016/j.ejcb.2011.01.010 21439673

[B19] Eckmann L. Jung H. C. Schürer-Maly C. Panja A. Morzycka-Wroblewska E. Kagnoff M. F. (1993). Differential cytokine expression by human intestinal epithelial cell lines: Regulated expression of interleukin 8. Gastroenterology 105 (6), 1689–1697. 10.1016/0016-5085(93)91064-o 8253345

[B20] Fang R. Veitch N. C. Kite G. C. Porter E. A. Simmonds M. S. (2013). Enhanced profiling of flavonol glycosides in the fruits of sea buckthorn (*Hippophae rhamnoides*). J. Agric. Food Chem. 61 (16), 3868–3875. 10.1021/jf304604v 23517173

[B21] Forrester S. J. Kikuchi D. S. Hernandes M. S. Xu Q. Griendling K. K. (2018). Reactive oxygen species in metabolic and inflammatory signaling. Circ. Res. 122 (6), 877–902. 10.1161/circresaha.117.311401 29700084 PMC5926825

[B22] Fuentes J. de Camargo A. C. Atala E. Gotteland M. Olea-Azar C. Speisky H. (2021). Quercetin oxidation metabolite present in onion peel protects Caco-2 cells against the oxidative stress, NF-kB activation, and loss of epithelial barrier function induced by NSAIDs. J. Agric. Food Chem. 69 (7), 2157–2167. 10.1021/acs.jafc.0c07085 33591188

[B23] Gong X. Ji M. Xu J. Zhang C. Li M. (2020). Hypoglycemic effects of bioactive ingredients from medicine food homology and medicinal health food species used in China. Crit. Rev. Food Sci. Nutr. 60 (14), 2303–2326. 10.1080/10408398.2019.1634517 31309854

[B24] Grefner N. M. Gromova L. V. Gruzdkov A. A. Komissarchik Y. Y. (2015). Interaction of glucose transporters SGLT1 and GLUT2 with cytoskeleton in enterocytes and Caco2 cells during hexose absorption. Cell Tiss. Biol. 9 (1), 45–52. 10.1134/S1990519X15010034

[B25] Gu Y. Wang X. Liu F. Zhang J. Zhang X. Liu J. (2022). Total flavonoids of sea buckthorn (*Hippophae rhamnoides* L.) improve MC903-induced atopic dermatitis-like lesions. J. Ethnopharmacol. 292, 115195. 10.1016/j.jep.2022.115195 35306042

[B26] Guo C. Han L. Li M. Yu L. (2020). Seabuckthorn (Hippophaë rhamnoides) freeze-dried powder protects against high-fat diet-induced obesity, lipid metabolism disorders by modulating the gut microbiota of mice. Nutrients 12 (1), 265. 10.3390/nu12010265 PMC7020008 31968607

[B27] Guo X. Yang B. Cai W. Li D. (2017). Effect of sea buckthorn (hippophae rhamnoides L.) on blood lipid profiles: A systematic review and meta-analysis from 11 independent randomized controlled trials. Trends Food Sci. Technol. 61, 1–10. 10.1016/j.tifs.2016.11.007

[B28] Han P. Gu J.-Q. Li L.-S. Wang X.-Y. Wang H.-T. Wang Y. (2021). The association between intestinal bacteria and allergic diseases-cause or consequence? Front. Cell. Infect. Microbiol. 11, 650893. 10.3389/fcimb.2021.650893 33937097 PMC8083053

[B29] Hilgers A. R. Conradi R. A. Burton P. S. (1990). Caco-2 cell monolayers as a model for drug transport across the intestinal mucosa. Pharm. Res. 7, 902–910. 10.1023/A:1015937605100 2235888

[B30] Hollman P. C. H. Bijsman M. N. C. P. van Gameren Y. Cnossen E. P. J. de Vries J. H. M. Katan M. B. (1999). The sugar moiety is a major determinant of the absorption of dietary flavonoid glycosides in man. Free Radic. Res. 31 (6), 569–573. 10.1080/10715769900301141 10630681

[B31] Hosoi T. Hirose R. Saegusa S. Ametani A. Kiuchi K. Kaminogawa S. (2003). Cytokine responses of human intestinal epithelial-like Caco-2 cells to the nonpathogenic bacterium *Bacillus subtilis* (natto). Int. J. Food Microbiol. 82 (3), 255–264. 10.1016/s0168-1605(02)00311-2 12593928

[B32] Jia Q. Zhang S. Zhang H. Yang X. Cui X. Su Z. (2020). A comparative study on polyphenolic composition of berries from the Tibetan plateau by UPLC‐Q‐orbitrap MS system. C&B 17 (4), e2000033. 10.1002/cbdv.202000033 32119759

[B33] Kammalla A. K. Ramasamy M. K. Chintala J. Dubey G. P. Agrawal A. Kaliappan I. (2015). Comparative pharmacokinetic interactions of Quercetin and Rutin in rats after oral administration of European patented formulation containing Hipphophae rhamnoides and Co-administration of Quercetin and Rutin. Eur. J. Drug Metab. Pharmacokinet. 40 (3), 277–284. 10.1007/s13318-014-0206-9 24888486

[B34] Kim S. Y. Jin C. Y. Kim C. H. Yoo Y. H. Choi S. H. Kim G. Y. (2019). Isorhamnetin alleviates lipopolysaccharide-induced inflammatory responses in BV2 microglia by inactivating NF-κB, blocking the TLR4 pathway and reducing ROS generation. Int. J. Mol. Med. 43 (2), 682–692. 10.3892/ijmm.2018.3993 30483725 PMC6317673

[B35] Kumar S. Dikshit M. (2019). Metabolic insight of neutrophils in health and disease. Front. Immunol. 10, 2099. 10.3389/fimmu.2019.02099 31616403 PMC6764236

[B36] Kumar Singh A. Cabral C. Kumar R. Ganguly R. Kumar Rana H. Gupta A. (2019). Beneficial effects of dietary polyphenols on gut microbiota and strategies to improve delivery efficiency. Nutrients 11 (9), 2216. 10.3390/nu11092216 PMC6770155 31540270

[B37] Kwon O. Eck P. Chen S. Corpe C. P. Lee J. H. Kruhlak M. (2007). Inhibition of the intestinal glucose transporter GLUT2 by flavonoids. FASEB J. 21 (2), 366–377. 10.1096/fj.06-6620com 17172639

[B38] Leelawat S. Leelawat K. (2018). Cytokine secretion of peripheral blood mononuclear cells by hydnocarpus anthelminthicus seeds. J. Trop. Med. 2018, 6854835. 10.1155/2018/6854835 29973956 PMC6008724

[B39] Li J. Wu R. Qin X. Liu D. Lin F. Feng Q. (2017). Isorhamnetin inhibits IL‑1β‑induced expression of inflammatory mediators in human chondrocytes. Mol. Med. Rep. 16 (4), 4253–4258. 10.3892/mmr.2017.7041 28731170

[B40] Li T. S. C. (1999). sea buckthorn: New crop opportunity. Alexandria, VA: ASHS Press.

[B41] Lopetuso L. R. Scaldaferri F. Bruno G. Petito V. Franceschi F. Gasbarrini A. (2015). The therapeutic management of gut barrier leaking: The emerging role for mucosal barrier protectors. Eur. Rev. Med. Pharmacol. Sci. 19, 1068–1076. 25855934

[B42] Ma X. Yang W. Kallio H. Yang B. (2022a). Health promoting properties and sensory characteristics of phytochemicals in berries and leaves of sea buckthorn (Hippophaë rhamnoides). Crit. Rev. Food Sci. Nutr. 62 (14), 3798–3816. 10.1080/10408398.2020.1869921 33412908

[B43] Ma Z. Sun Q. Chang L. Peng J. Zhang M. Ding X. (2022b). A natural anti-obesity reagent derived from sea buckthorn polysaccharides: Structure characterization and anti-obesity evaluation *in vivo* . Food Chem. 375, 131884. 10.1016/j.foodchem.2021.131884 34953239

[B44] Melzig M. F. Loose R. (1998). Inhibition of lipopolysaccharide (LPS)-induced endothelial cytotoxicity by selected flavonoids. Planta Med. 64 (5), 397–399. 10.1055/s-2006-957467 9690338

[B45] Mlcek J. Jurikova T. Skrovankova S. Sochor J. (2016). Quercetin and its anti-allergic immune response. Molecules 21 (5), 623. 10.3390/molecules21050623 PMC6273625 27187333

[B46] Mohammad S. Thiemermann C. (2020). Role of metabolic endotoxemia in systemic inflammation and potential interventions. Front. Immunol. 11, 594150. 10.3389/fimmu.2020.594150 33505393 PMC7829348

[B47] Mulati A. Zhang X. Zhao T. Ren B. Wang L. Liu X. (2021). Isorhamnetin attenuates high-fat and high-fructose diet induced cognitive impairments and neuroinflammation by mediating MAPK and NFκB signaling pathways. Food Funct. 12 (19), 9261–9272. 10.1039/d0fo03165h 34606526

[B48] Murphy E. A. Velazquez K. T. Herbert K. M. (2015). Influence of high-fat diet on gut microbiota. Curr. Opin. Clin. Nutr. Metabolic Care 18 (5), 515–520. 10.1097/MCO.0000000000000209 PMC4578152 26154278

[B49] Nalbantsoy A. Karabay-Yavasoglu N. U. Deliloglu-Gurhan I. (2011). Determination of *in vivo* toxicity and *in vitro* cytotoxicity of lipopolysaccharide isolated from *Salmonella* Enteritidis and its potential use for production of polyclonal antibody. Food Agric. Immunol. 22 (3), 271–281. 10.1080/09540105.2011.569883

[B50] Neethi Raj P. Shaji B. V. Haritha V. H. Anie Y. (2018). Neutrophil secretion modulates neutrophil and monocyte functions during hyperglucose and/or hyperinsulin conditions *in vitro* . J. Cell. Immunother. 4 (2), 65–70. 10.1016/j.jocit.2018.02.001

[B51] Nikolaus S. Bauditz J. Gionchetti P. Witt C. Lochs H. Schreiber S. (1998). Increased secretion of pro-inflammatory cytokines by circulating polymorphonuclear neutrophils and regulation by interleukin 10 during intestinal inflammation. Gut 42 (4), 470–476. 10.1136/gut.42.4.470 9616306 PMC1727082

[B52] Nishio K. Horie M. Akazawa Y. Shichiri M. Iwahashi H. Hagihara Y. (2013). Attenuation of lipopolysaccharide (LPS)-induced cytotoxicity by tocopherols and tocotrienols. Redox Biol. 1 (1), 97–103. 10.1016/j.redox.2012.10.002 24024142 PMC3757666

[B53] Olas B. Skalski B. (2022). Preparations from various organs of sea buckthorn (*Elaeagnus rhamnoides* (L.) A. Nelson) as important regulators of hemostasis and their role in the treatment and prevention of cardiovascular diseases. Nutrients 14 (5), 991. 10.3390/nu14050991 35267966 PMC8912734

[B54] Olas B. (2018). The beneficial health aspects of sea buckthorn (Elaeagnus rhamnoides (L.) A.Nelson) oil. J. Ethnopharmacol. 213, 183–190. 10.1016/j.jep.2017.11.022 29166576

[B55] Olszewska M. A. Granica S. Kolodziejczyk-Czepas J. Magiera A. Czerwińska M. E. Nowak P. (2020). Variability of sinapic acid derivatives during germination and their contribution to antioxidant and anti-inflammatory effects of broccoli sprouts on human plasma and human peripheral blood mononuclear cells. Food Funct. 11 (8), 7231–7244. 10.1039/D0FO01387K 32760968

[B56] Opal S. M. Scannon P. J. Vincent J. L. White M. Carroll S. F. Palardy J. E. (1999). Relationship between plasma levels of lipopolysaccharide (LPS) and LPS-binding protein in patients with severe sepsis and septic shock. J. Infect. Dis. 180 (5), 1584–1589. 10.1086/315093 10515819

[B57] Peluso M. R. Miranda C. L. Hobbs D. J. Proteau R. R. Stevens J. F. (2010). Xanthohumol and related prenylated flavonoids inhibit inflammatory cytokine production in LPS-activated THP-1 monocytes: Structure-activity relationships and *in silico* binding to myeloid differentiation protein-2 (MD-2). Planta Med. 76 (14), 1536–1543. 10.1055/s-0029-1241013 20309792

[B58] Pundir S. Garg P. Dviwedi A. Ali A. Kapoor V. K. Kapoor D. (2021). Ethnomedicinal uses, phytochemistry and dermatological effects of *Hippophae rhamnoides* L.: A review. J. Ethnopharmacol. 266, 113434. 10.1016/j.jep.2020.113434 33017636

[B59] Ruszczyński M. Urbańska M. Szajewska H. (2014). Gelatin tannate for treating acute gastroenteritis: A systematic review. Ann. Gastroenterol. 27 (2), 121–124. 24733622 PMC3982626

[B60] Ryter S. W. Kim H. P. Hoetzel A. Park J. W. Nakahira K. Wang X. (2007). Mechanisms of cell death in oxidative stress. Antioxidants Redox Signal. 9 (1), 49–89. 10.1089/ars.2007.9.49 17115887

[B61] Sampath V. P. (2018). Bacterial endotoxin-lipopolysaccharide; structure, function and its role in immunity in vertebrates and invertebrates. Agric. Nat. Resour. 52, 115–120. 10.1016/j.anres.2018.08.002

[B62] Seo K. Yang J. H. Kim S. C. Ku S. K. Ki S. H. Shin S. M. (2014). The antioxidant effects of isorhamnetin contribute to inhibit COX-2 expression in response to inflammation: A potential role of HO-1. Inflammation 37 (3), 712–722. 10.1007/s10753-013-9789-6 24337631

[B63] Serreli G. Melis M. P. Corona G. Deiana M. (2019). Modulation of LPS-induced nitric oxide production in intestinal cells by hydroxytyrosol and tyrosol metabolites: Insight into the mechanism of action. Food Chem. Toxicol. 125, 520–527. 10.1016/j.fct.2019.01.039 30735752

[B64] Shi H. Ter Horst R. Nielen S. Bloemendaal M. Jaeger M. Joosten I. (2022). The gut microbiome as mediator between diet and its impact on immune function. Sci. Rep. 12, 5149. 10.1038/s41598-022-08544-y 35338162 PMC8956630

[B65] Siegień J. Buchholz T. Popowski D. Granica S. Osińska E. Melzig M. F. (2021). Pancreatic lipase and α-amylase inhibitory activity of extracts from selected plant materials after gastrointestinal digestion *in vitro* . Food Chem. 355, 129414. 10.1016/j.foodchem.2021.129414 33773461

[B66] Singh V. Yang B. Choudhary S. Mörsel J.-T. Zubarev Y. A. Mohini K. Sharma V. K. (2014). Seabuckthorn (Hippophae L.): a multipurpose wonder plant. New Delhi: Daya Publishing House.

[B67] Singleton V. L. Orthofer R. Lamuela-Raventós R. M. (1999). [14] Analysis of total phenols and other oxidation substrates and antioxidants by means of folin-ciocalteu reagent. Methods Enzym. 299, 152–178. 10.1016/s0076-6879(99)99017-1

[B68] Sireswar S. Biswas S. Dey G. (2020). Adhesion and anti-inflammatory potential of *Lactobacillus rhamnosus* GG in a sea buckthorn based beverage matrix. Food Funct. 11 (3), 2555–2572. 10.1039/c9fo02249j 32154524

[B69] Świerczewska A. Buchholz T. Melzig M. F. Czerwińska M. E. (2019). *In vitro* α-amylase and pancreatic lipase inhibitory activity of Cornus mas L. and Cornus alba L. fruit extracts. J. Food Drug Anal. 27 (1), 249–258. 10.1016/j.jfda.2018.06.005 30648578 PMC9298612

[B70] Teshika J. D. Zakariyyah A. M. Zaynab T. Zengin G. Rengasamy K. R. Pandian S. K. (2019). Traditional and modern uses of onion bulb (*Allium cepa* L.): A systematic review. Crit. Rev. Food Sci. Nutr. 59, S39–S70. 10.1080/10408398.2018.1499074 30040448

[B71] Tkacz K. Wojdyło A. Turkiewicz I. P. Bobak Ł. Nowicka P. (2019). Anti-oxidant and anti-enzymatic activities of sea buckthorn (Hippophaë rhamnoides L.) fruits modulated by chemical components. Antioxidants (Basel) 8 (12). 10.3390/antiox8120618 PMC6943611 31817215

[B72] Tucureanu M. M. Rebleanu D. Constantinescu C. A. Deleanu M. Voicu G. Butoi E. (2018). Lipopolysaccharide-induced inflammation in monocytes/macrophages is blocked by liposomal delivery of Gi-protein inhibitor. Int. J. Nanomedicine 13, 63–76. 10.2147/ijn.S150918 PMC5743190 29317816

[B73] Vomhof-Dekrey E. E. Picklo M. J. Sr. (2012). The nrf2-antioxidant response element pathway: A target for regulating energy metabolism. J. Nutr. Biochem. 23 (10), 1201–1206. 10.1016/j.jnutbio.2012.03.005 22819548

[B74] Wang X. Liu J. Zhang X. Zhao S. Zou K. Xie J. (2018). Seabuckthorn berry polysaccharide extracts protect against acetaminophen induced hepatotoxicity in mice *via* activating the Nrf-2/HO-1-SOD-2 signaling pathway. Phytomedicine 38, 90–97. 10.1016/j.phymed.2017.11.007 29425659

[B75] Wells J. M. Brummer R. J. Derrien M. MacDonald T. T. Troost F. Cani P. D. (2017). Homeostasis of the gut barrier and potential biomarkers. Am. J. Physiol. Gastrointest. Liver Physiol. 312 (3), G171–G193. 10.1152/ajpgi.00048.2015 27908847 PMC5440615

[B76] Wen L. Jiang Y. Yang J. Zhao Y. Tian M. Yang B. (2017). Structure, bioactivity, and synthesis of methylated flavonoids. Ann. N. Y. Acad. Sci. 1398 (1), 120–129. 10.1111/nyas.13350 28436044

[B77] Wéra O. Lancellotti P. Oury C. (2016). The dual role of neutrophils in inflammatory bowel diseases. Jcm 5 (12), 118. 10.3390/jcm5120118 PMC5184791 27999328

[B78] Xie Y. Duan J. Fu Q. Xia M. Zhang L. Li G. (2015). Comparison of isorhamnetin absorption properties in total flavones of *Hippophae rhamnoides* L. with its pure form in a Caco-2 cell model mediated by multidrug resistance-associated protein. Eur. J. Pharm. Sci. 73, 1–8. 10.1016/j.ejps.2015.03.008 25813735

[B79] Xu Y. Li J. Lin Z. Liang W. Qin L. Ding J. (2022). Isorhamnetin alleviates airway inflammation by regulating the Nrf2/Keap1 pathway in a mouse model of COPD. Front. Pharmacol. 13. 10.3389/fphar.2022.860362 PMC8988040 35401244

[B80] Yamamoto Y. He P. Klein T. W. Friedman H. (1994). Endotoxin induced cytotoxicity of macrophages is due to apoptosis caused by nitric oxide production. J. Endotoxin Res. 1 (3), 181–187. 10.1177/096805199400100307

[B81] Zeng M. Y. Inohara N. Nuñez G. (2017). Mechanisms of inflammation-driven bacterial dysbiosis in the gut. Mucosal Immunol. 10 (1), 18–26. 10.1038/mi.2016.75 27554295 PMC5788567

[B82] Zhang W. Zhao J. Zhu X. Zhuang X. Pang X. Wang J. (2010). Antihyperglycemic effect of aqueous extract of sea buckthorn (hippophae rhamnoides L.) seed residues in streptozotocin-treated and high fat-diet-fed rats. J. Food Biochem. 34, no. 10.1111/j.1745-4514.2010.00337.x

[B83] Zhao H. Kong L. Shao M. Liu J. Sun C. Li C. (2022). Protective effect of flavonoids extract of Hippophae rhamnoides L. on alcoholic fatty liver disease through regulating intestinal flora and inhibiting TAK1/p38MAPK/p65NF-κB pathway. J. Ethnopharmacol. 292, 115225. 10.1016/j.jep.2022.115225 35341932

[B84] Zheng Y. Scow J. S. Duenes J. A. Sarr M. G. (2012). Mechanisms of glucose uptake in intestinal cell lines: Role of GLUT2. Surgery 151 (1), 13–25. 10.1016/j.surg.2011.07.010 21943636 PMC3237888

