# Supplementary material for: Fruits of Hippophaë rhamnoides in human leukocytes and Caco-2 cell monolayer models—A question about their preventive role in lipopolysaccharide leakage and cytokine secretion in endotoxemia
Source: Front Pharmacol. 2022 Sep 30;13:981874. doi: 10.3389/fphar.2022.981874 (PMC9561609; doi:10.3389/fphar.2022.981874)
Supplement: Supplementary file 1 [file DataSheet1.pdf]

Supplementary Material

1 Chemicals

Acetonitrile HiPerSolv Chromanorm® (20060.320) was purchased from VWR Chemicals (Radnor, PA, USA). HPLC grade methanol (106009) was purchased from Merck (Darmstadt, Germany). Dimethyl sulfoxide (DMSO, 113635509) was purchased from Chempur (Piekary Śląskie, Poland). Water was obtained using a Millipore Simfilter Simplicity UV (Molsheim, France) water purification system. Chloroform, diethyl ether, petroleum ether, ethyl acetate (EtOAc), ethanol (EtOH), methanol (MeOH), *n*-hexane, *n*-butanol for extraction were obtained from POCh (Gliwice, Poland). Formic acid (HCOOH) for use as an additive in the UHPLC-MS eluent was purchased from Sigma-Aldrich Chemie GmbH (Steinheim, Germany).

2 Isolation procedures

The isolation of compounds was performed in the Soxhlet apparatus according to the scheme presented in Figure S1. The fractions were pooled based on their TLC profile [silica gel F254; ethyl acetate-acetic acid-formic acid-water (100:11:11:26; *v*/*v*/*v*), derivatized with 1% methanolic solution of aminoethyl ester of diphenylboronic acid (Naturstoffreagenz A) at the temperature of 95 °C].

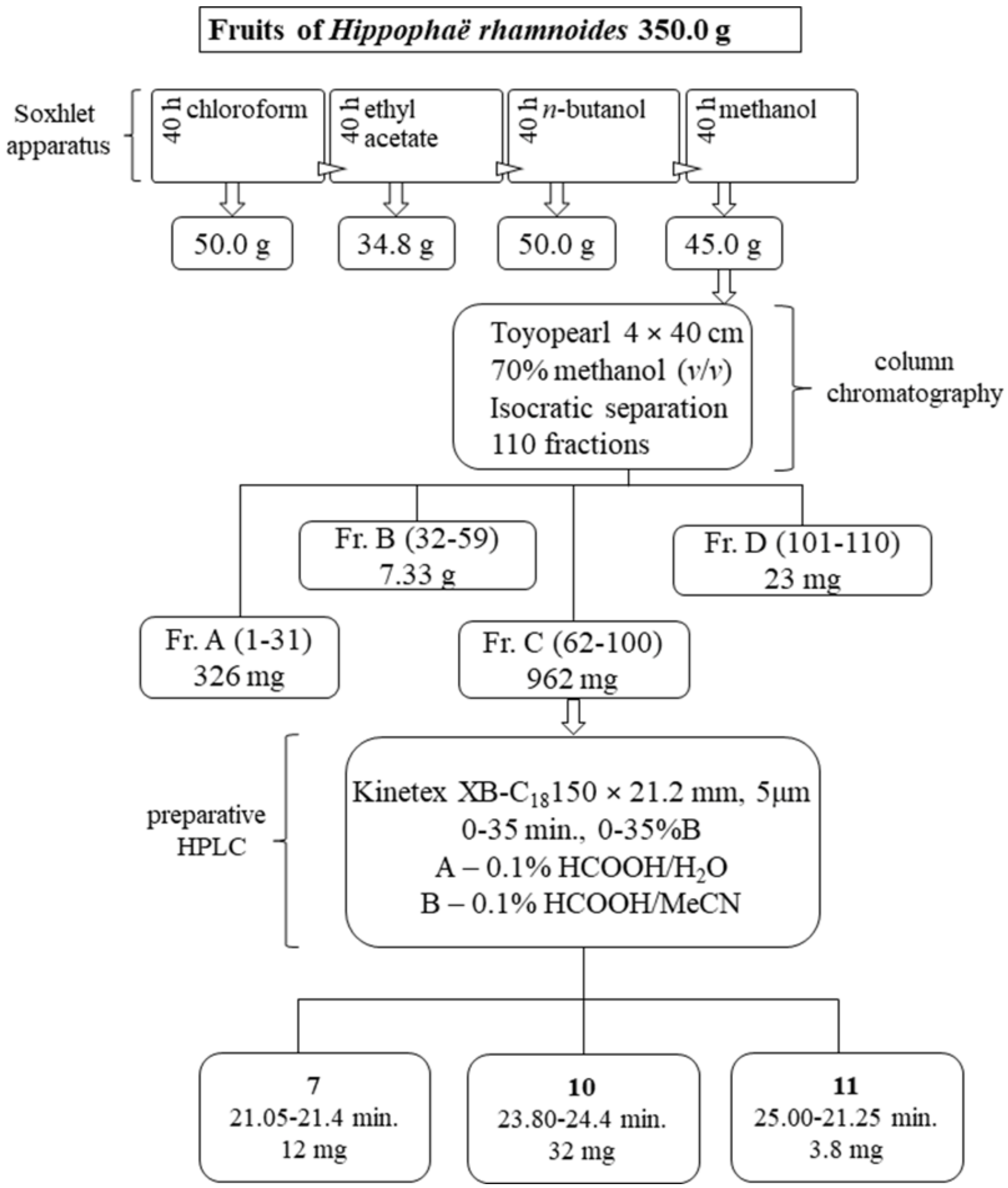

Supplementary Figure S1. The scheme of isolation of compounds from the fruit of *Hippophaë rhamnoides.*

3 Chromatographic analysis

Chromatographic analysis was performed on a UHPLC-3000 RS system (Dionex, Germany) with DAD and an AmaZon SL ion trap mass spectrometer with an ESI interface (Bruker Daltonik GmbH, Germany). Separations were performed on a Kinetex XB-C18 column (150 × 2.1 mm, 1.7 µm) (Phenomenex, USA). The column oven temperature was 25 °C. For preliminary phytochemical analyses of the extracts and fractions, mobile phase A was 0.1% HCOOH in water, and mobile phase B was 0.1% HCOOH in acetonitrile. The gradient program was as follows: 0 – 20 min. 4 – 26% B; 20 – 60 min. 26 – 95% B. The flow rate was 0.2 mL/min. The column was equilibrated for 10 min. between

injections. UV spectra were recorded in the range of 200 – 800 nm, and chromatograms were acquired at 240, 280, 325 nm, or 520 nm. The LC eluate was introduced directly into the ESI interface without splitting. The nebulizer pressure was 40 psi; the dry gas flow was 9 L/min; the oven temperature was 300 °C, and the capillary voltage was 4.5 kV. Analyses were carried out scanning from *m*/*z* 200 to 2,200. Compounds were analyzed in negative and positive ion modes. The MS^2 fragmentation patterns were obtained for the most abundant ion (Świerczewska et al., 2019).

4 Identification of compounds

The structures of the isolated compounds were determined based on NMR spectral analysis. The identity of the aglycone and the absolute configuration of the sugars were confirmed based on the analysis of hydrolysates and sugar derivatization. NMR spectra were recorded on a Bruker DSX-400 spectrophotometer at the Laboratory of Nuclear Magnetic Resonance (NMR) Spectroscopy at the Department of Analytical Chemistry and Biomaterials, Medical University of Warsaw.

5 Hydrolysis

The accurately weighted 1-3 mg of glycoside was treated with 5% HCl in a water bath for 2 h at a temperature of 90 °C. Next, the reaction mixture was extracted with 3 × 3mL diethyl ether. The organic fraction was allowed to evaporate in a thermoblock at a temperature of 30 °C. A sample for HPLC analysis was prepared by dissolving in 250 µL of methanol. The aqueous fraction was concentrated under reduced pressure. The hydrolyzate was dissolved in 1 mL of distilled water, next 1 mL of methanolic (*S*)-(-)-*α*-methylbenzylamine solution (10 mg/mL), and 1 mL of methanolic $NaBH_3CN$ solution (3 mg/mL) were added. The reaction mixture was placed in a 40 °C water bath for 4h, then acidified to pH 3-4 with glacial acetic acid and evaporated under reduced pressure. The portion (1.2-1.4 mL) of acetic anhydride and pyridine (1:1, *v*/*v*) was added to the oily residue. After 24 h at room temperature, excess reagents were removed from the reaction mixture by washing it 5 times with portions of water (ca. 1 mL) and evaporated under reduced pressure. An aliquot of the oily residue was suspended in water (2 mL) and extracted with chloroform (3 mL) three times. The content of the vial was allowed to evaporate in a thermoblock at 50 °C. A sample for HPLC analysis was prepared by dissolving it in acetonitrile-water (20:80, *v*/*v*).

Compound 7

Isorhamnetin 3-*O*-*β*-D-glucopyranosyl-7-*O*-*α*-L-rhamnoside

The structure of compound 7 was confirmed by a comparison of NMR data with available literature (Rösch et al., 2004).

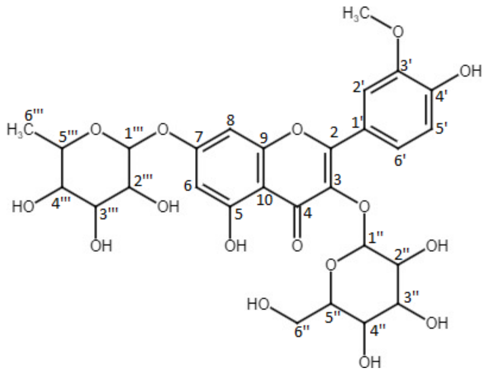

Supplementary Figure S2. The structure of compound 7.

a) ^{1}H NMR

^{1}H NMR (500 MHz, DMSO) δ 7.94 (d, *J* = 2.1 Hz, H-2'), 7.56 (dd, *J* = 8.4, 2.1 Hz, H-6'), 6.91 (d, *J* = 7.2 Hz, H-5'), 6.83 (d, *J* = 2.1 Hz, H-8), 6.45 (d, *J* = 2.1 Hz, H-6), 5.57 (d, *J* = 7.4 Hz, H-1''), 5.55 (s, H-1'''), 3.83 (s, 3'-OCH3), 1.10 (d, *J* = 6.2 Hz, H-6''').

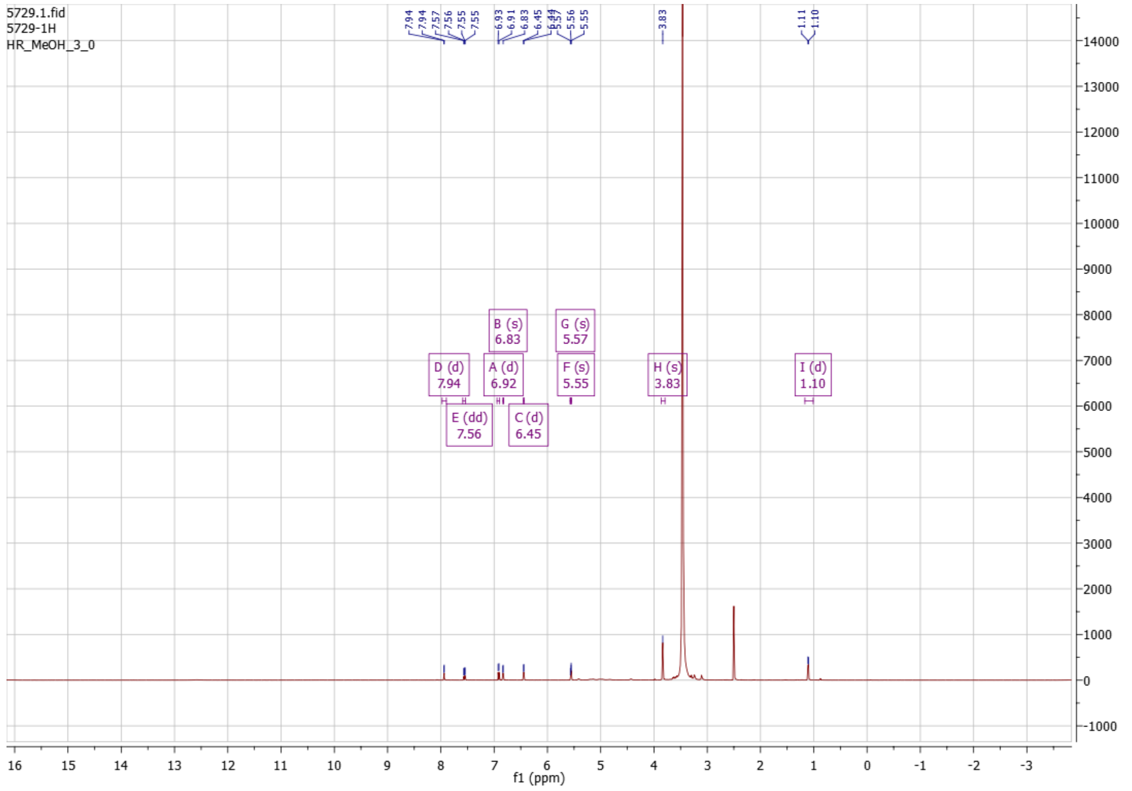

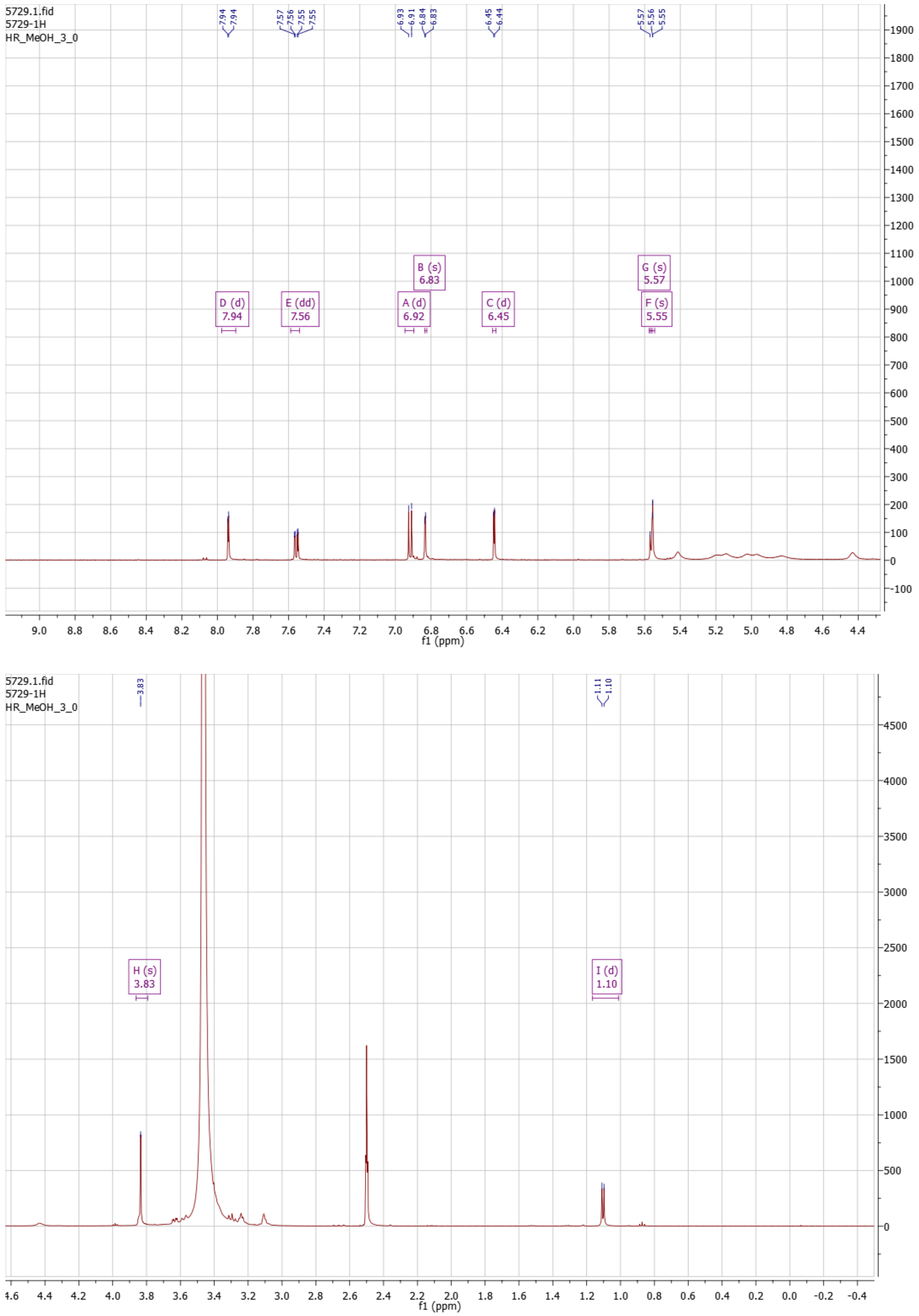

Supplementary Figure S3. ^{1}H NMR spectrum of compound 7.

b) **^{13}C NMR**

^{13}C NMR (126 MHz, DMSO) δ 177.67 (C-4), 161.68 (C-7), 160.98 (C-5), 156.96 (C-2), 156.10 (C-9), 149.84 (C-3'), 147.07 (C-4'), 133.36 (C-3), 122.44 (C-6'), 120.93 (C-1'), 115.36 (C-5'), 113.52 (C-2'), 105.79 (C-10), 100.80 (C-1''), 99.50 (C-6), 98.40 (C-1'''), 94.71 (C-8), 77.59 (C-5''), 76.50 (C-3''), 74.43 (C-2''), 71.72 (C-4'''), 70.34 (C-3'''), 70.17 (C-4''), 69.92 (C-5'''), 60.72 (C-6''), 55.81 (C3'-OCH_3), 18.03 (C-6''').

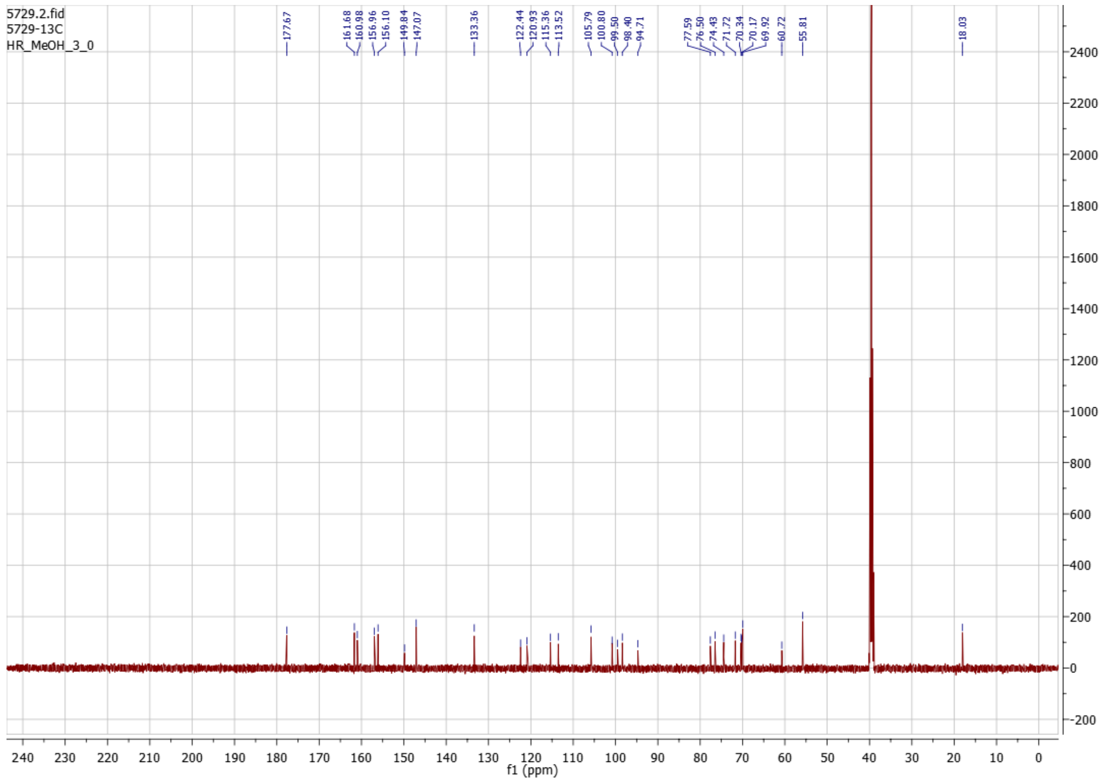

Supplementary Figure S4. ^{13}C NMR spectrum of compound 7.

Compound 10

Isorhamnetin 3-*O*-*α*-L-rhamnopyranosyl-(1→6)-*β*-D-glucoside

The structure of compound 10 was confirmed by a comparison of NMR data with available literature (Fukunaga et al., 1988; Su et al., 2008; Znati et al., 2014).

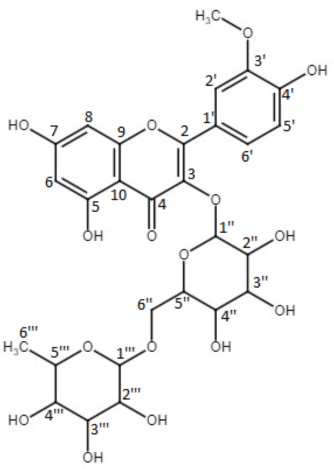

Supplementary Figure S5. The structure of compound 10.

a) ^{1}H NMR

^{1}H NMR (300 MHz, DMSO) δ 12.57 (s, 5-OH), 7.85 (d, *J* = 1.8 Hz, H-2'), 7.52 (dd, *J* = 8.4, 1.9 Hz, H-6'), 6.91 (d, *J* = 9.2 Hz, H-5'), 6.43 (d, *J* = 1.9 Hz, H-8), 6.20 (d, *J* = 1.9 Hz, H-6), 5.40 (d, *J* = 19.6, 5.1 Hz, H-1''), 5.10 (m, H-1'''), 3.83 (s, 3'-OCH_3), 3.78 – 3.06 (m, overlapped, sugar protons), 0.97 (d, *J* = 6.1 Hz, H-6''').

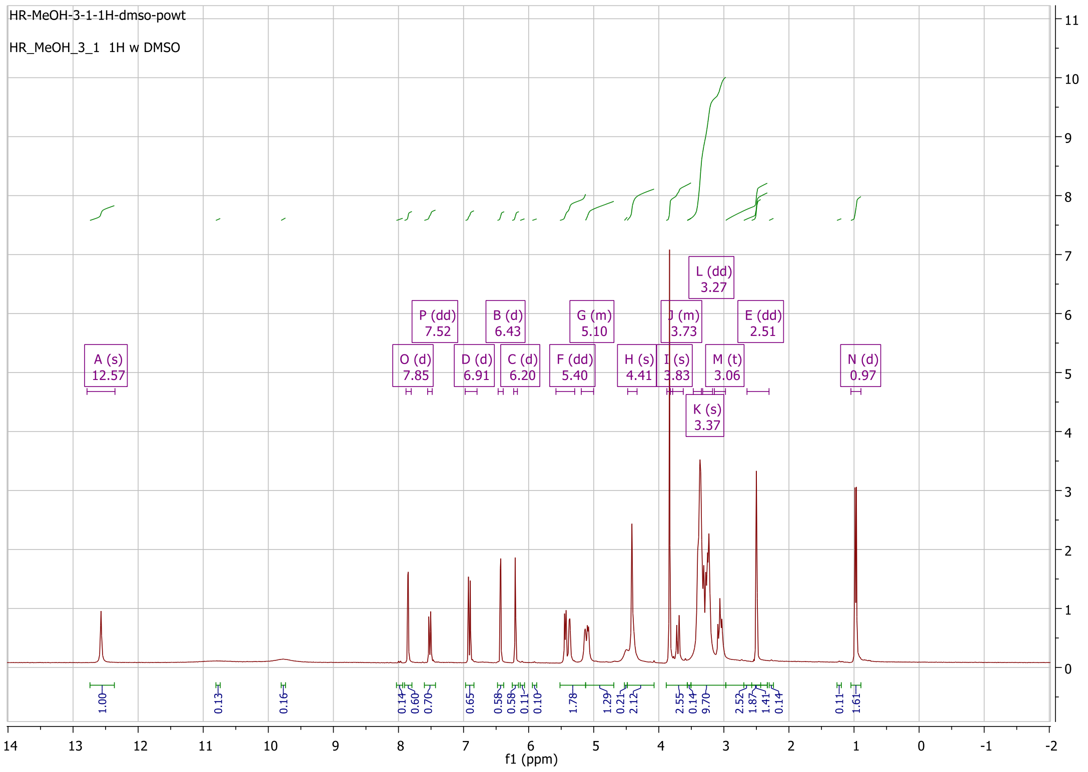

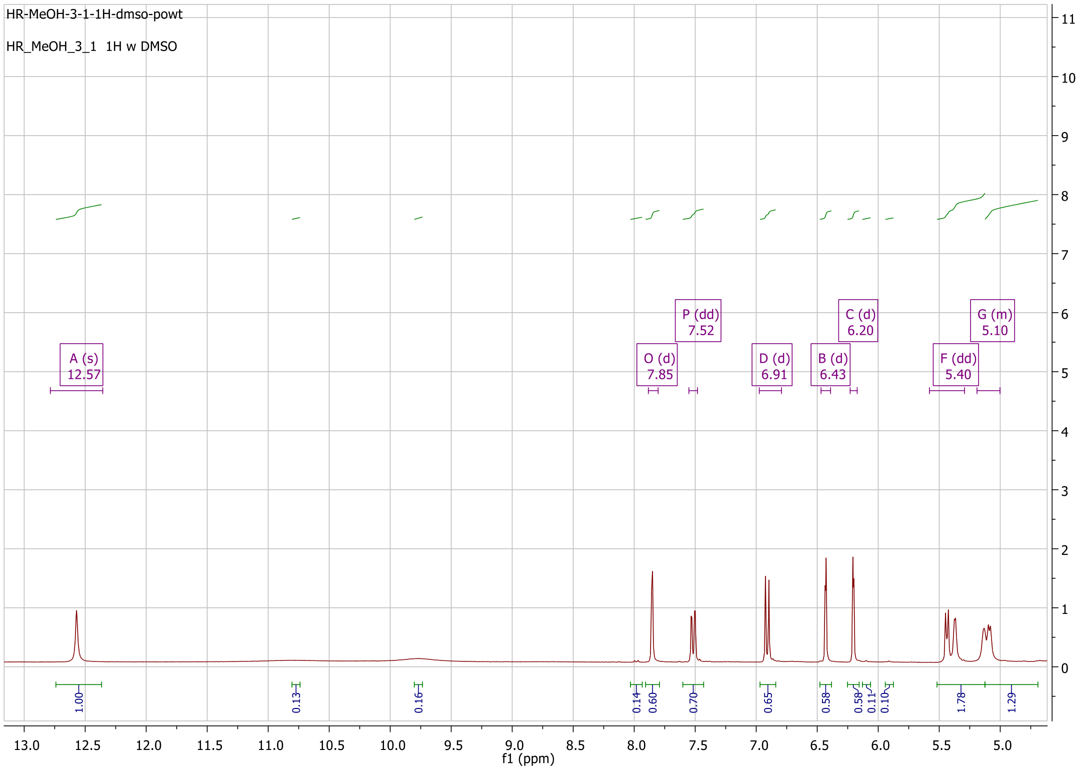

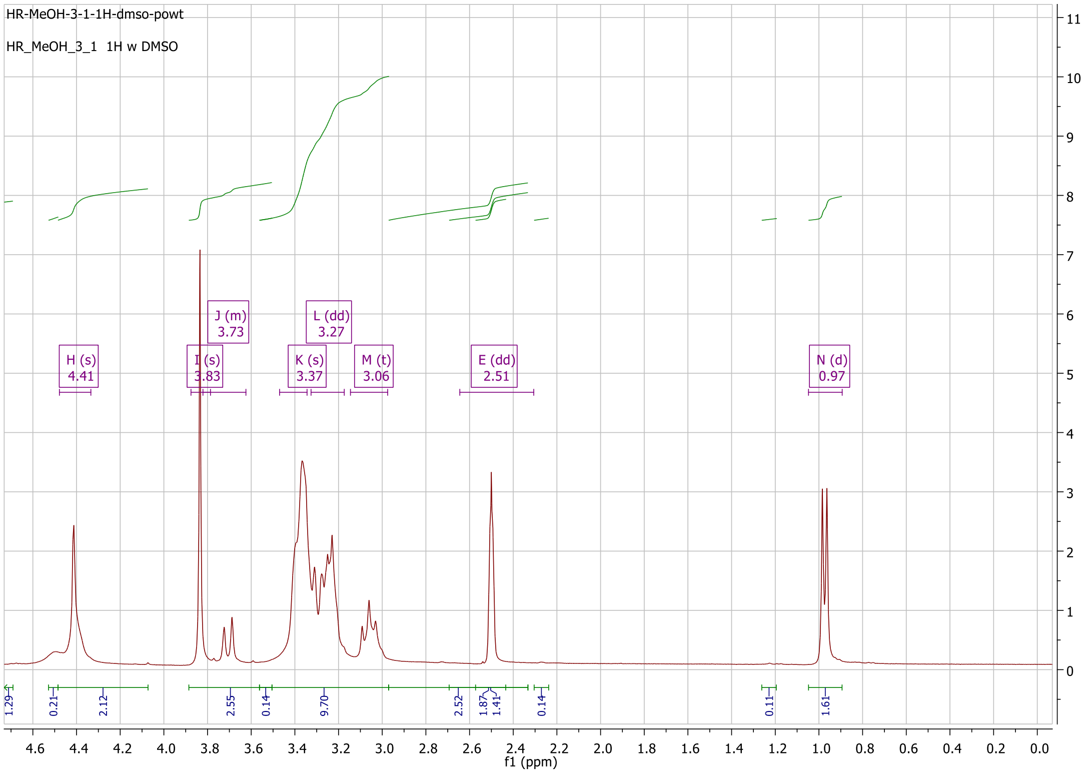

Supplementary Figure S6. ^{1}H NMR spectrum of compound 10.

b) ^{13}C NMR

^{13}C NMR (75 MHz, DMSO) δ 177.78 (C-4), 164.59 (C-7), 161.63 (C-5), 156.91 (C-2), 149.84 (C-3'), 147.33 (C-4'), 133.46 (C-3), 122.72 (C-6'), 121.49 (C-1'), 115.69 (C-5'), 113.72 (C-2'), 104.46 (C-10), 101.63 (C-1''), 101.35 (C-1'''), 99.18 (C-6), 94.25 (C-8), 76.84 (C-5''), 76.38 (C-3''), 74.73 (C-2''), 72.24 (C-4'''), 71.04 (C-4''), 70.76 (C-2'''), 70.54 (C-3'''), 68.74 (C-5'''), 67.29 (C-6''), 56.11 (C3'-OCH_3), 18.16 (C-6''').

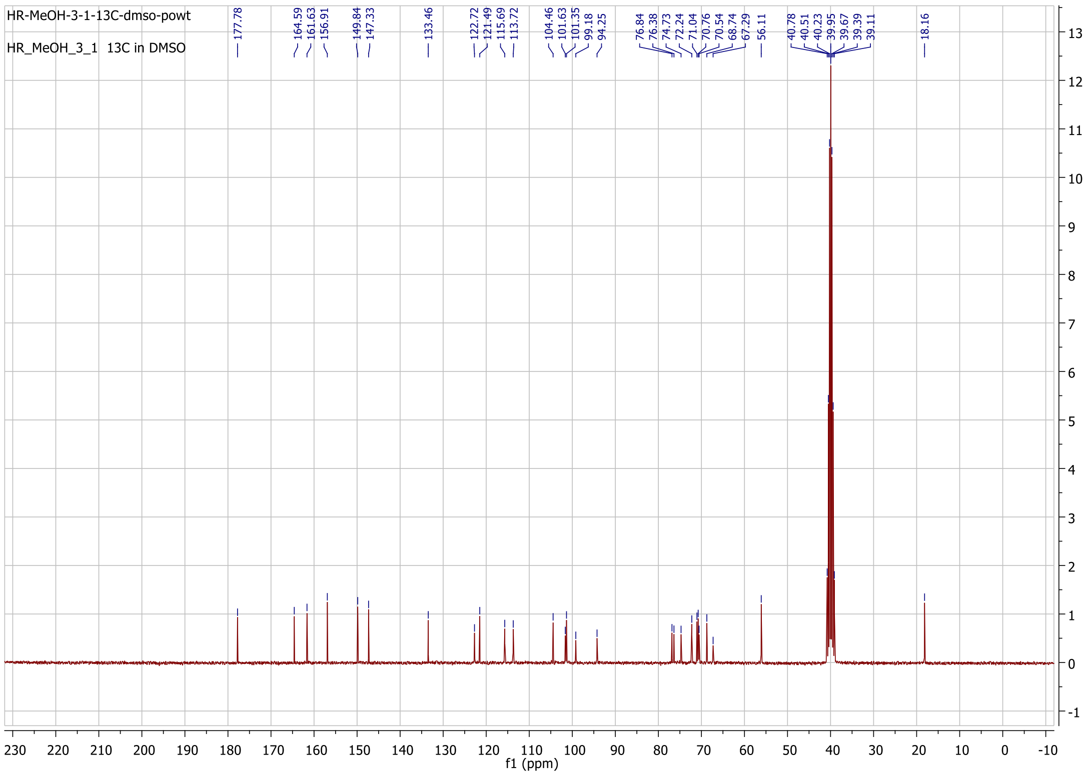

Supplementary Figure S7. ^{13}C NMR spectrum of compound 10.

c) absolute configuration of sugar residue

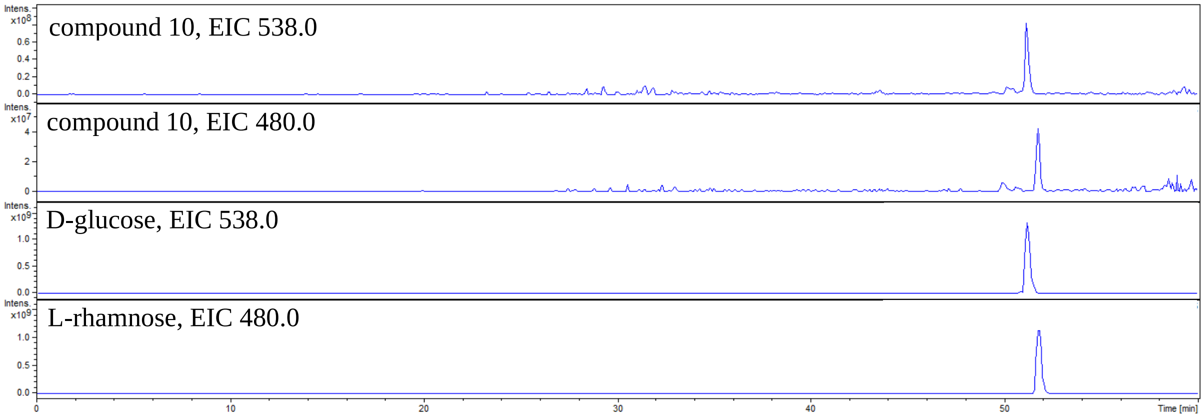

Supplementary Figure S8. Ion chromatograms for *m/z* 480 and *m/z* 538 in the negative ESI mode.

Compound 11

Isorhamnetin 3-*O*-glucoside

The structure of compound 11 was confirmed by a comparison of NMR data with available literature (Su et al., 2008).

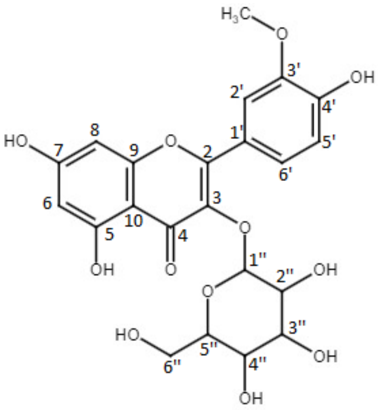

Supplementary Figure S9. The structure of compound 11.

a) ^{1}H NMR

^{1}H NMR (300 MHz, DMSO) δ 7.94 (d, *J* = 1.9 Hz, H-2'), 7.50 (dd, *J* = 8.2 Hz, 2.0 Hz, H-6'), 6.91 (d, *J* = 8.5 Hz, H-5'), 6.44 (d, *J* = 2.0 Hz, H-8), 6.21 (d, *J* = 1.9 Hz, H-6), 5.57 (d, *J* = 7.6 Hz, H-1''), 3.84 (s, 3'-OCH_3), 3.52 – 3.00 (m, overlapped, sugar protons).

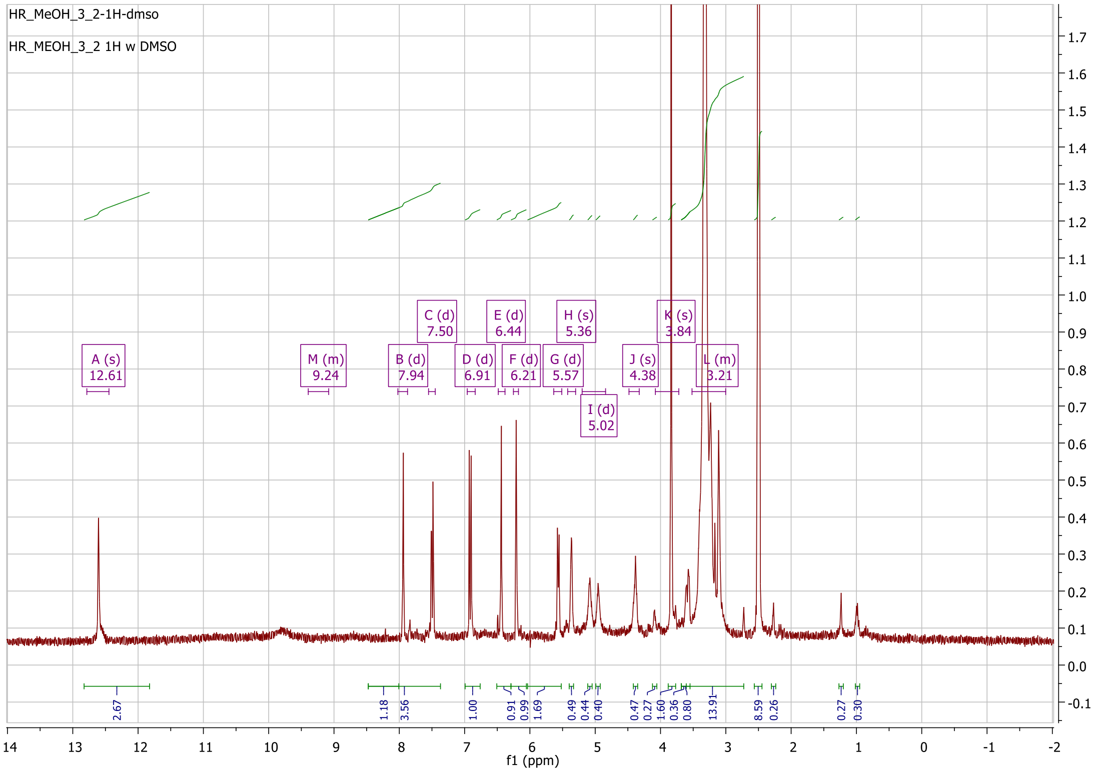

HR_MeOH_3_2-1H-dmso
HR_MEOH_3_2 1H w DMSO
A (s) 12.61
M (m) 9.24
B (d) 7.94
C (d) 7.50
D (d) 6.91
E (d) 6.44
F (d) 6.21
G (d) 5.57
H (s) 5.36
I (d) 5.02
J (s) 4.38
K (s) 3.84
L (m) 3.21
2.67
1.18
3.56
1.00
0.91
0.99
1.69
0.49
0.44
0.40
0.47
0.27
1.60
0.36
0.80
13.91
8.59
0.26
0.27
0.30
f1 (ppm)

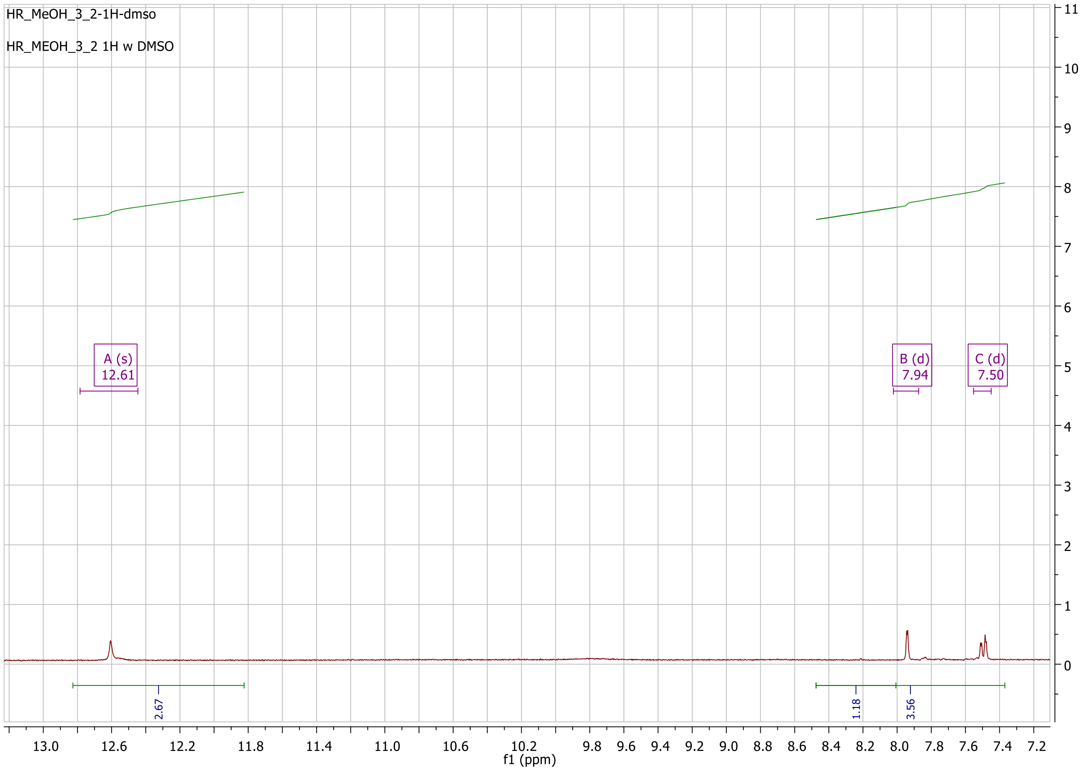

HR_MeOH_3_2-1H-dmso
HR_MEOH_3_2 1H w DMSO
A (s) 12.61
B (d) 7.94
C (d) 7.50
2.67
1.18
3.56
f1 (ppm)

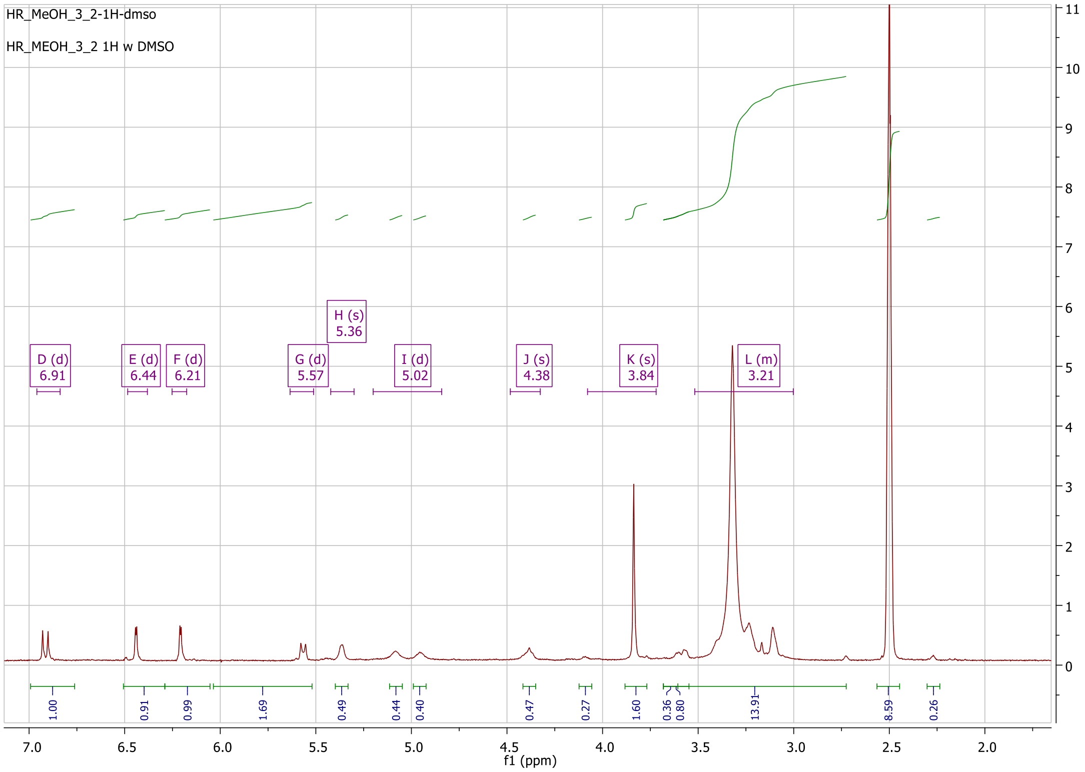

Supplementary Figure S10. ^{1}H NMR spectrum of compound 11.

b) ^{13}C NMR

^{13}C NMR (75 MHz, DMSO) δ 177.84 (C-4), 164.70 (C-7), 161.68 (C-5), 156.84 (C-2), 156.71 (C-9), 149.85 (C-3'), 147.33 (C-4'), 133.41 (C-3), 121.53 (C-1'), 115.66 (C-5'), 113.94 (C-2'), 104.46 (C-10), 101.22 (C-1''), 76.87 (C-3''), 74.79 (C-2''), 56.13 (C3'-OCH_3).

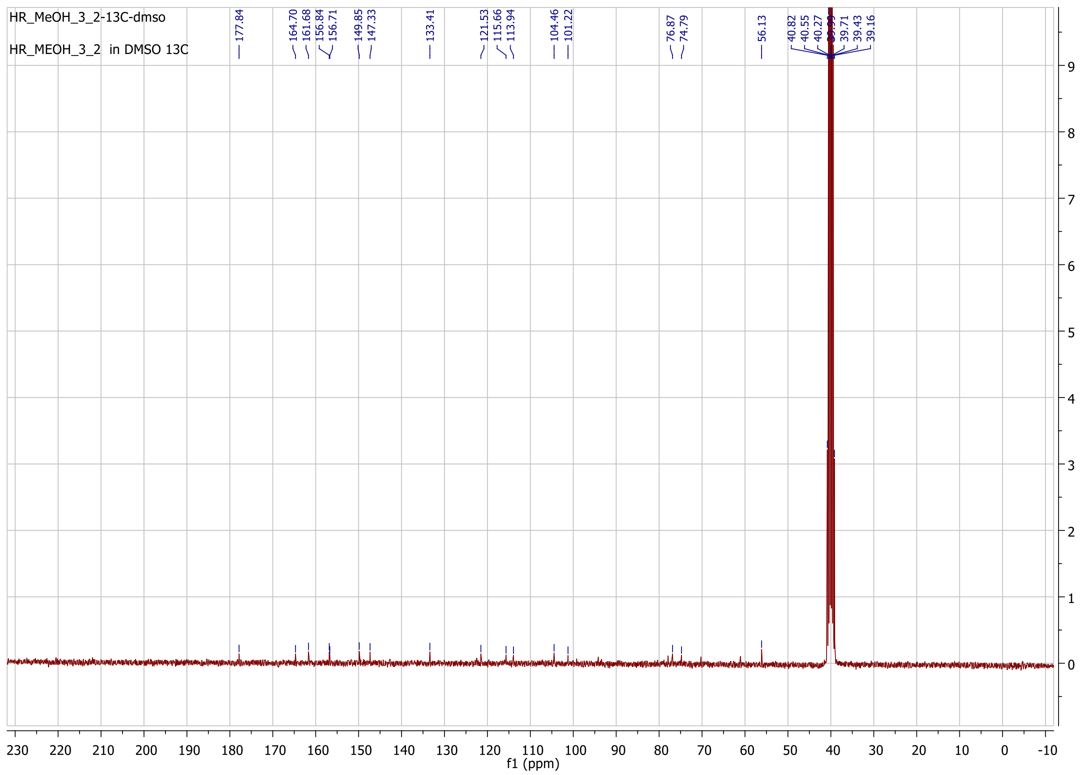

Supplementary Figure S11. ^{13}C NMR spectrum of compound 11.

c) absolute configuration of sugar residues

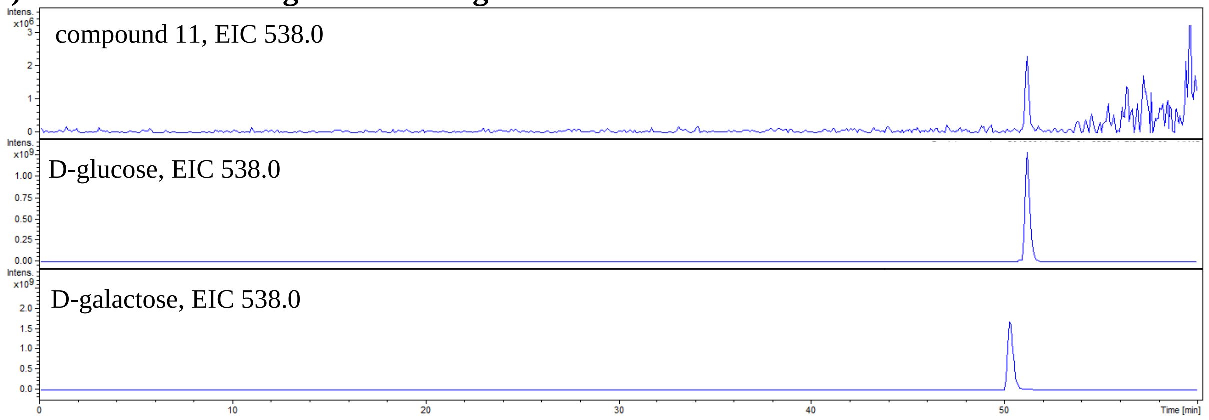

Supplementary Figure S12. Ion chromatograms for *m/z* 538 in the negative ESI mode.

d) identification of aglycone

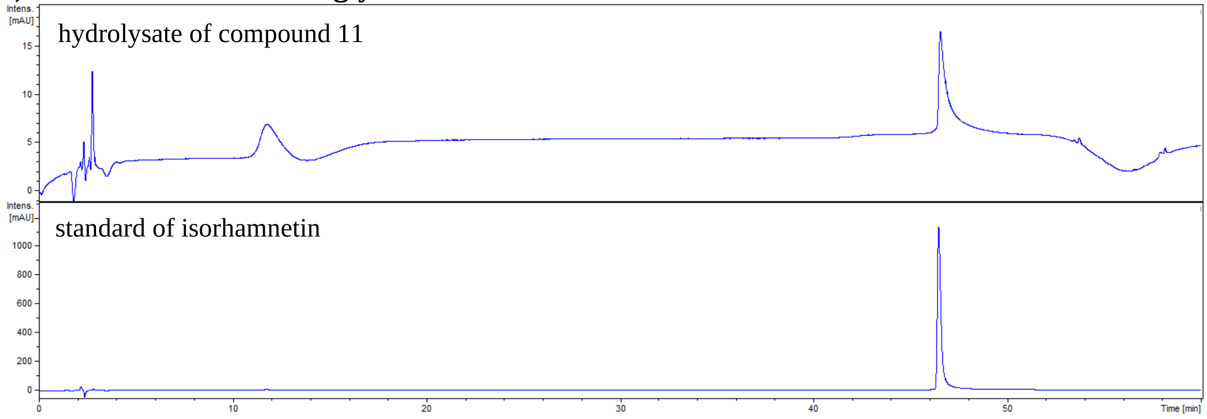

Supplementary Figure S13. HPLC-DAD chromatogram hydrolysate of isorhamnetin 3-*O*-glucoside (11) and the standard of isorhamnetin.

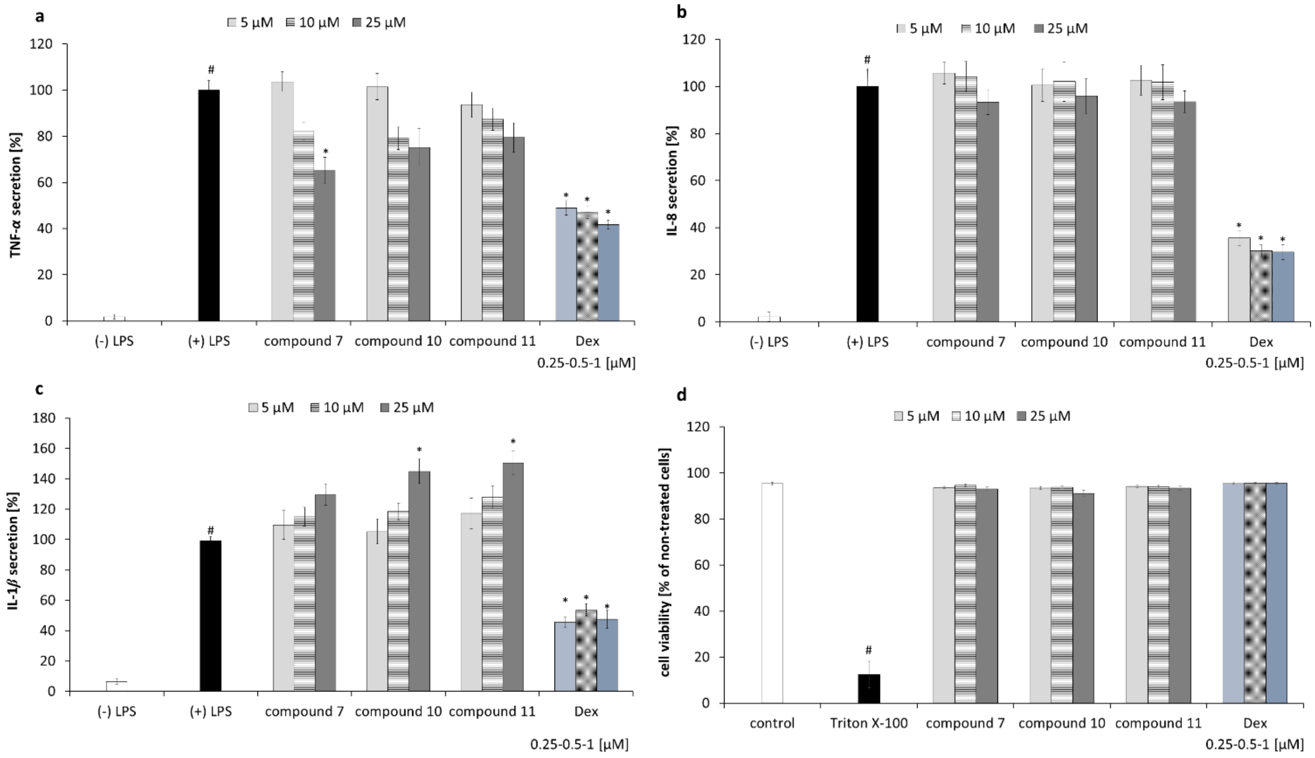

Supplementary Figure S14. Effect of isolated compounds on secretion of TNF-*α* (a), IL-8 (b), and IL-1*β* (c) by PMN and cell viability (d) (mean ± SEM [%]); Dex – dexamethasone. #$p < 0.001$ vs. (−) LPS ; *$p < 0.05$ vs. (+) LPS

References

Fukunaga, T., Kajikawa, I., Nishiya, K., Watanabe, Y., Suzuki, N., Takeya, K., et al. (1988). Studies on the Constituents of the European Mistletoe, Viscum album L. II. *Chemical and Pharmaceutical Bulletin* 36(3)**,** 1185-1189. doi: 10.1248/cpb.36.1185.

Rösch, D., Krumbein, A., Mügge, C., and Kroh, L.W. (2004). Structural investigations of flavonol glycosides from sea buckthorn (*Hippophae rhamnoides*) pomace by NMR spectroscopy and HPLC-ESI-MS^n. *Journal of Agricultural and Food Chemistry* 52**,** 4039-4046. doi: 10.1021/jf0306791.

Su, X.C., Chen, L., and Aisa, H.A. (2008). Flavonoids and sterols from *Alhagi sparsifolia*. *Chemistry of Natural Compounds* 3**,** 365. doi: 10.1007/s10600-008-9064-5.

Świerczewska, A., Buchholz, T., Melzig, M.F., and Czerwińska, M.E. (2019). In vitro α-amylase and pancreatic lipase inhibitory activity of *Cornus mas* L. and *Cornus alba* L. fruit extracts. *Journal of Food and Drug Analysis* 27(1)**,** 249-258. doi: 10.1016/j.jfda.2018.06.005.

Znati, M., Ben Jannet, H., Cazaux, S., Souchard, J.P., Harzallah Skhiri, F., and Bouajila, J. (2014). Antioxidant, 5-lipoxygenase inhibitory and cytotoxic activities of compounds isolated from the *Ferula lutea* flowers. *Molecules* 19(10)**,** 16959-16975. doi: 10.3390/molecules191016959.
